# Bottom-up Assembly of the Phytochrome Network

**DOI:** 10.1371/journal.pgen.1006413

**Published:** 2016-11-07

**Authors:** Maximiliano Sánchez-Lamas, Christian D. Lorenzo, Pablo D. Cerdán

**Affiliations:** 1 Fundación Instituto Leloir, IIBBA-CONICET, Buenos Aires, Argentina; 2 Facultad de Ciencias Exactas y Naturales, Universidad de Buenos Aires, Buenos Aires, Argentina; University of Lausanne, SWITZERLAND

## Abstract

Plants have developed sophisticated systems to monitor and rapidly acclimate to environmental fluctuations. Light is an essential source of environmental information throughout the plant’s life cycle. The model plant *Arabidopsis thaliana* possesses five phytochromes (phyA-phyE) with important roles in germination, seedling establishment, shade avoidance, and flowering. However, our understanding of the phytochrome signaling network is incomplete, and little is known about the individual roles of phytochromes and how they function cooperatively to mediate light responses. Here, we used a bottom-up approach to study the phytochrome network. We added each of the five phytochromes to a phytochrome-less background to study their individual roles and then added the phytochromes by pairs to study their interactions. By analyzing the 16 resulting genotypes, we revealed unique roles for each phytochrome and identified novel phytochrome interactions that regulate germination and the onset of flowering. Furthermore, we found that ambient temperature has both phytochrome-dependent and -independent effects, suggesting that multiple pathways integrate temperature and light signaling. Surprisingly, none of the phytochromes alone conferred a photoperiodic response. Although phyE and phyB were the strongest repressors of flowering, both phyB and phyC were needed to confer a flowering response to photoperiod. Thus, a specific combination of phytochromes is required to detect changes in photoperiod, whereas single phytochromes are sufficient to respond to light quality, indicating how phytochromes signal different light cues.

## Author Summary

As sessile organisms, plants respond to and integrate environmental information. An intriguing aspect is how plants integrate this information. We studied the interactions among members of the phytochrome family of photoreceptors, which detect the changes in light quality that occur upon shading by other plants, as well as the duration of daylength, which indicates seasonal changes. We conducted these studies in Arabidopsis, which bears five phytochromes (phyA-phyE). We show that the individual roles of each phytochrome (in the absence of others) are different but, more importantly, that their combinations give different properties to the system. phyE, for instance, regulates flowering in a temperature-dependent manner, indicating that phyE signaling is a point of integration between light and temperature cues. phyC is poorly active on its own, but it is essential for the phytochrome-dependent photoperiodic flowering. In long-days (mimicking late spring) phyC promotes flowering indirectly, by inhibiting phyB and phyE signaling, which are themselves repressors of flowering. Conversely, under the short days of winter phyC becomes a flowering repressor because phyB requires phyC only for this specific response. Therefore, phyC is essential for the detection of photoperiod by phytochromes and suggest a conserved role for phyC in the photoperiodism of angiosperms.

## Introduction

Plant photoreceptor signaling networks are sensitive to a large dynamic range of light inputs. Plant light signaling systems are sensitive enough to induce germination in response to extremely short exposures of light, as encountered during soil tillage, and very low light intensities, as experienced under soil litter, and yet able to detect subtle variations in light quality under full sunlight or changes in photoperiod. These abilities depend partly on the existence of multiple photoreceptor families with differential spectral properties and on the sub-functionalization of photoreceptor family members, which resulted in the emergence of photoreceptors with distinct properties and the capacity to interact to modulate light sensitivity [1, 2].

In the model plant Arabidopsis (*Arabidopsis thaliana*), thirteen different sensory photoreceptors have been characterized to date, with absorption spectra ranging from UV-B (280 nm) to far-red light (730 nm) (FR) [3]. The phytochromes are a family of red light (R) and FR photoreceptors consisting of five members (phyA-phyE) [4], and plant development under natural conditions depends on their ability to toggle between the Pr (inactive) form, which absorbs R, and the Pfr (active) form, which absorbs FR. The phytochromes are synthesized in the Pr form and photoconverted to the Pfr form after absorption of R. FR can then convert Pfr back to Pr. Thus, the proportion of phytochromes in the Pfr form is a function of the R to FR ratio. As plants absorb R for photosynthesis, but reflect FR, a decrease in the R/FR ratio indicates the presence of neighboring vegetation, which at some point may compete for light resources [5]. Shade-intolerant plants respond to low R/FR ratios by elongating their stems and petioles, to outcompete their neighbors, and accelerating flowering, to ensure their reproductive success. These responses are known collectively as the shade avoidance syndrome (SAS) [1]. Phytochromes regulate plant development throughout the life cycle, from germination to flowering. For instance, phytochromes promote germination by stimulating gibberellin (GA) synthesis and sensitivity [6, 7], promote deetiolation during early seedling development, inhibit hypocotyl and stem elongation by altering auxin levels [8], entrain the circadian clock, and regulate flowering time [2].

Phytochromes exist as homodimers or heterodimers, which are translocated to the nucleus upon photoconversion to the Pfr form [9]. Upon light exposure during seedling establishment, phytochromes alter the expression of thousands of genes [1, 2, 10]. They induce these complex responses by interacting with members of a family of basic-helix-loop-helix (bHLH) transcription factors, the PHYTOCHROME INTERACTING FACTORS (PIF). The PIFs are repressors of germination and deetiolation and are degraded by proteasomes upon interaction with phytochromes [1, 2, 7].

The roles of phytochromes were mostly studied in the model systems Arabidopsis and rice (*Oryza sativa*), using photoreceptor mutants [11–17]. Given their similar spectral properties, it is not possible to dissect the roles of each phytochrome in plant development without using genetic tools. Studies have mainly analyzed single phytochrome mutants, and few have examined higher order mutant combinations [2]. Although these studies have been fundamental in establishing which phytochromes contribute to which developmental responses, they failed to pinpoint which particular phytochrome activates which developmental program or pathway. Further, cross-talk has been reported to exist between phytochrome signaling pathways [15, 18, 19]; however, it is unclear if the identified interacting phytochromes are sufficient for the interaction or if other phytochromes are also required. Increased complexity may also be expected, because heterodimers form among phytochrome family members [20, 21]. In studies in which phytochromes were overexpressed, other phytochromes were present in the genetic background, raising questions as to whether the signal was generated by single or multiple phytochromes [22–25].

To resolve these issues, we used a bottom-up approach, similar to that employed by Coen and Meyerowitz to generate their ABC model of plant flower development [26]. They used a mutant background devoid of the three kinds of flower development genes (A, B, and C) to which they “added” each gene separately and then by pairs to dissect the roles of each gene and to decipher the mutual interactions that led to flower development. Using a similar approach, we obtained the whole set of quadruple and triple phytochrome mutants in the same genetic background (i.e., Columbia). In this way, we “added” each phytochrome alone and each possible pair of phytochromes to a phytochrome-less background to study the effects of each individual phytochrome in isolation and to examine all pair wise interactions, both direct and indirect. Our work reveals the distinct roles of each phytochrome and identifies how the phytochromes interact with each other, thereby revealing novel properties of the phytochrome signaling system that are necessary for regulating the photoperiodic response.

## Results

### Individual roles and binary phytochrome interactions in promoting germination

**Fig 1:**
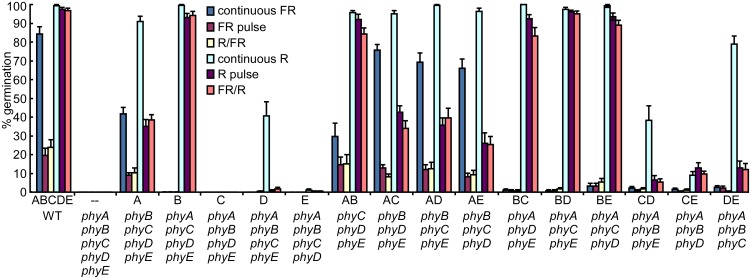
The effect of single phytochrome photoreceptors on the regulation of germination and their synergistic interactions. Germination of the genotypes indicated on the abscissas at 23°C under different light regimes (for clarity, phytochromes present in each line are indicated above, in capital letters, and genotypes below, in italics). Light regimes: continuous FR (60 μmol m^-2^ s^-1^), continuous red (R) (30 μmol m^-2^ s^-1^), a 15-min R pulse, a 15-min FR pulse, a 15-min R pulse followed immediately by a 15-min FR pulse (R/FR), a 15-min FR pulse followed immediately by a 15-min R pulse (FR/R), and darkness. Data are averages ± SE of 16 independent plates with 20 seeds each and 4 independent seed pools (collected from independently grown plants).

After obtaining the whole set of phytochrome quadruple and triple mutants in the Columbia background, we evaluated their germination under different light conditions. Under continuous white light (WL), phyA, phyB, or phyD alone was sufficient to induce germination, phyE was a poor inducer of germination, and phyC did not induce germination. The quintuple phytochrome mutant failed to germinate under WL (S1 Fig). Under R and FR treatments, only phyB produced a R/FR reversible response, while phyA produced a response to continuous FR and to a single FR pulse (the so-called very low fluence response, VLFR) (Fig 1), as expected [18]. As under WL, phyC did not induce germination at all under continuous FR or FR pulses and phyE only rarely did so. The simplest explanation for the low activity of phyC is the inability of the PHYC apoprotein to accumulate in the absence of phyB [20, 27] (S2 Fig). On the other hand, phyD promoted germination in response to continuous R but not to single R pulses (Fig 1). The requirement of continuous R by phyD is consistent with previous observations in phyD overexpressing lines which also required continuous R to inhibit hypocotyl elongation [24].

Triple mutant combinations allowed us to study phytochrome interactions. Despite its negligible role as an inducer of germination when present alone, phyE acted synergistically with phyC and with phyD to induce germination after exposure to continuous R or to a single pulse of R (Fig 1). Further, phyE acted synergistically with phyB at low R:FR ratios (i.e., during FR treatments), consistent with previous reports [6, 19]. phyC, phyD, and phyE interacted synergistically with phyA under continuous FR, a treatment that specifically activates phyA [18].

When we evaluated the GA sensitivity of the various mutants, a different picture emerged. phyB and phyA were the most important positive regulators of GA sensitivity (S1 Table, S3A and S3C Fig) and were both antagonized by phyC, phyD, and phyE (S3A Fig). These results indicate that phyC, phyD, and phyE not only promote germination by acting synergistically (Fig 1), but also antagonize germination by reducing the effects of phyA and phyB on GA sensitivity (S3A and S3C Fig). This “gas and brake” behavior could be important for regulating GA signaling homeostasis once seeds germinate, as GA and light antagonize each other during seedling emergence [1].

As mentioned above, accumulation of PHYC apoprotein depends on phyB (S2 Fig) [20, 27]. We also found increased PHYA levels in the presence of phyC, phyD or phyE in light-grown seedlings (S2 Fig). Therefore, the synergistic interactions in the promotion of germination may be explained, to some extent, by increased photoreceptor levels. Conversely, the antagonistic interactions that decrease GA sensitivity suggest more specific roles for phyC, phyD, and phyE.

### phyE and phyB repress flowering in long days in a temperature-dependent manner

**Fig 2:**
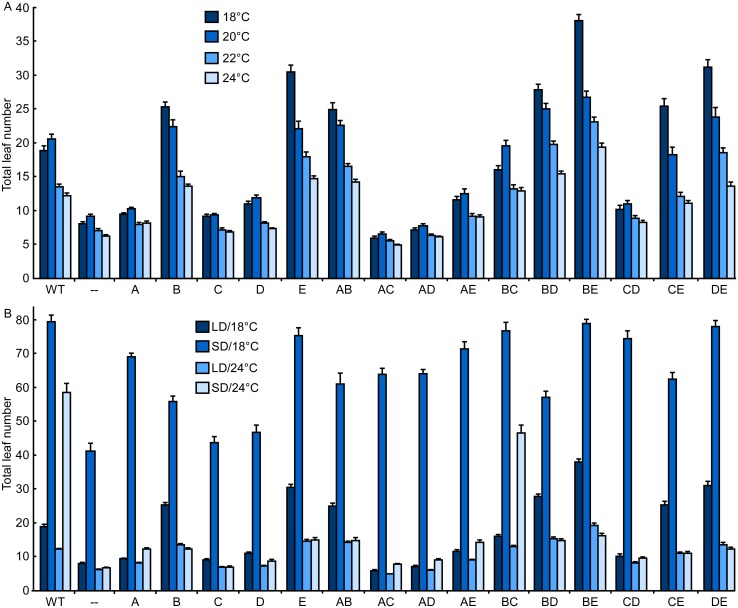
A network of phytochrome interactions is necessary to regulate flowering in response to photoperiod and temperature. Plants bearing the indicated phytochromes were grown under long days (LD, 16 h light/8 h dark) (A) or LD and short days (SD, 8 h light/16 h dark) (B), at temperatures ranging from 18 to 24°C. LD data in (B) are the same as in (A) and included for the purpose of direct comparison. The total leaf number at the time of flowering was recorded. Data points represent the mean ±SE of at least 18 plants from two independent experiments for each genotype and condition.

To evaluate how each phytochrome pathway influences flowering and is influenced by temperature, we measured flowering time for all genotypes under long-day (LD) conditions, at temperatures of 18 to 24°C (Fig 2A and S4A Fig). The quintuple phytochrome mutant was poorly responsive within this range of temperatures (slope: -0,365±0,217 leaves/°C). Surprisingly, phyE was found to be the strongest repressor of flowering and its effect was stronger at lower temperatures (slope: -2,580±0,429 leaves/°C Fig 2A). A similar effect was observed for phyB (slope: -2,121±0,389 leaves/°C Fig 2A). The temperature-dependent effects of phyE and phyB underscore the interaction between the light and temperature signaling pathways (Fig 2A). Conversely, phyD was a weak flowering repressor under all conditions tested, showing that phyD and phyE have distinct roles, with the former being more effective in promoting germination and the latter more effective in influencing flowering (Figs 1 and 2).

### phyA and phyC antagonize the repressive effect of phyE and phyB

phyA behaved as a weak repressor of flowering under LD at all temperatures tested (Fig 2A and S4A Fig). However, when combined with other phytochromes, phyA emerged as a strong antagonist, mainly by antagonizing phyE signaling. The presence of phyA eliminated most of the temperature responsiveness of plants bearing phyE alone, but not phyB, suggesting that signaling downstream phyE might be different, at least in part, from that downstream of phyB, and that some temperature effects might also be specific to phyE. phyA also antagonized the relatively weak role of phyD as a flowering repressor. These data suggest that the well-known role of phyA in promoting flowering [28] results, at least in part, from its antagonism of phyE and phyD signaling (Fig 2). This phyA effect is unlikely to be due to lower PHYD or PHYE levels in the presence of phyA (S2 Fig).

phyC had negligible effects when present alone. Similar to phyA, phyC antagonized the action of phyE, but contrary to phyA, also antagonized phyB at the lower temperatures (Fig 2A and S4A Fig). Taken together, these results emphasize the importance of both positive and negative phytochrome interactions in achieving a WT response to both photoperiod and temperature. Finally, when only phyA and phyC were present, plants flowered slightly earlier than the quintuple phytochrome mutant and plants bearing only phyA or phyC, suggesting a novel interaction between phyA and phyC leading to flowering promotion (Fig 2A and S4A Fig).

### Low ambient temperature represses flowering in short days in phytochrome-dependent and -independent manners

The flowering behavior of plants bearing phyE and phyA revealed that phytochrome signaling was strongly influenced by ambient temperature (Fig 2A and S4A Fig). In addition, the absence of phytochromes in the quintuple phytochrome mutant significantly reduced the sensitivity to temperature in LD conditions (Fig 2A) and the photoperiodic response was absent at 24°C (Fig 2B and S4B Fig). Surprisingly, in short days (SD) the quintuple phytochrome mutant flowered much later at 18°C compared to 24°C, showing that temperature also regulates flowering in a phytochrome-independent manner (Fig 2B and S4B Fig). Nevertheless, individual phytochromes also contributed to flowering repression in SD at 18°C. phyE was again the most efficient repressor, followed by phyA and phyB. phyD showed only weak effects on its own, whereas phyC effects were negligible. As in LD, phyC acted antagonistically to phyE, but contrary to LD, in SD at 18°C phyC acted synergistically with phyB and phyD to repress flowering.

Noteworthy, the quintuple phytochrome mutant responded to photoperiod only at 18°C, but not at 24°C (Fig 2B, see S5 Fig for the photoperiod effect). These results underscore the importance of interactions between temperature and phytochrome signaling in the control of flowering, but also show that there is at least one temperature responsive pathway that is phytochrome independent (Fig 2B and S4A Fig).

### phyC and phyB interactions are essential for photoperiod detection at 24°C

**Fig 3:**
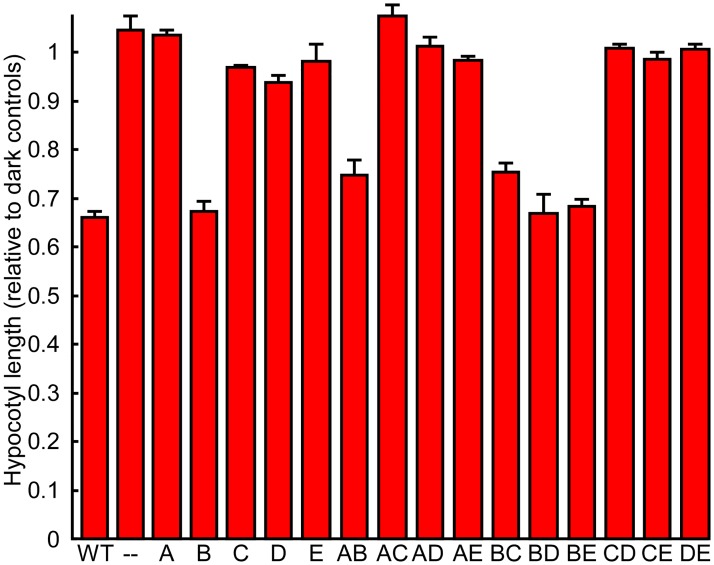
phyB is sufficient for a full hypocotyl response to R. Plants bearing the indicated phytochromes were stratified for 3 days at 4°C in the dark in a solution of 100 μM GA_4+7_ and then plated on MS salts agar plates and incubated at 23°C either under continuous red (R) light (20 μmol m^-2^ s^-1^) or kept in darkness (control) for 5 days. The values obtained under R are given relative to the corresponding dark control in each independent experiment. Data are averages ± SE of four independent plates.

To evaluate the photoperiodic response of the mutants, we compared the flowering time of plants grown in LD and SD conditions at either 18 or 24°C (Fig 2B, S5 Fig). At 24°C, none of the phytochromes conferred a photoperiodic response when present alone. Even phyB, which has roles in photoperiodic responses that have been extensively studied in single mutant analyses [29], failed to confer a photoperiodic response under the conditions tested. Genotypes bearing phyA showed a weak photoperiodic response; phyA behaved as a weak flowering repressor under SD conditions (Fig 2B). Interestingly, only the combination of phyB and phyC produced a strong photoperiodic response at 24°C (Fig 2B, S5 Fig). phyC and phyB form heterodimers and phyC requires phyB in Arabidopsis and rice [21, 30]. However, our results show that both phyB and phyC are required to confer a photoperiodic response, suggesting that the phyB/phyC heterodimer may have a specific and important role. Further, this specificity seems to be essential for the photoperiodic response, but not for the hypocotyl response to R (Fig 3), since phyB was sufficient to restore a WT response to R on its own, whereas other phytochromes promoted only subtle phenotypic changes in response to R (Fig 3). Consistent with a role for the phyB-phyC pair in photoperiodism, this phytochrome pair was the most effective in inhibiting hypocotyl elongation under SD and LD, but not in response to blue light (S6 Fig). These results underscore the role of phyC in the photoperiodic response, which changes from a flowering promoter under LD conditions (by antagonizing phyB and phyE; Fig 2A) to a flowering repressor under SD conditions (by acting in combination with phyB; Fig 2B).

### Phytochrome roles mainly depend on intrinsic properties

The differential effects of each phytochrome could be due to differences in the intrinsic properties of each photoreceptor or in the mRNA expression levels, translatability or distribution patterns. The intrinsic properties include the differential capacity to accumulate at the protein level, to heterodimerize or to signal to downstream factors, and the photochemical properties of each phytochrome. To rule out the effects of mRNA expression and distribution patterns, we generated transgenic lines in which each phytochrome fused to the hemaglutinin (HA) tag was driven by the 35S constitutive promoter in the quintuple phytochrome mutant background. At least eight independent transgenic lines for each phytochrome were obtained without previous selection for the phenotype other than herbicide resistance. Individual lines were evaluated for the flowering and germination phenotypes (S7 Fig).

The use of the same epitope tag allowed us a direct comparison of the levels of PHY apoprotein accumulation in each transgenic line (S8 Fig). Only one out of fourteen phyC lines expressed detectable levels, which is consistent with the phyC dependence on phyB (S2 Fig) [21].

**Fig 4:**
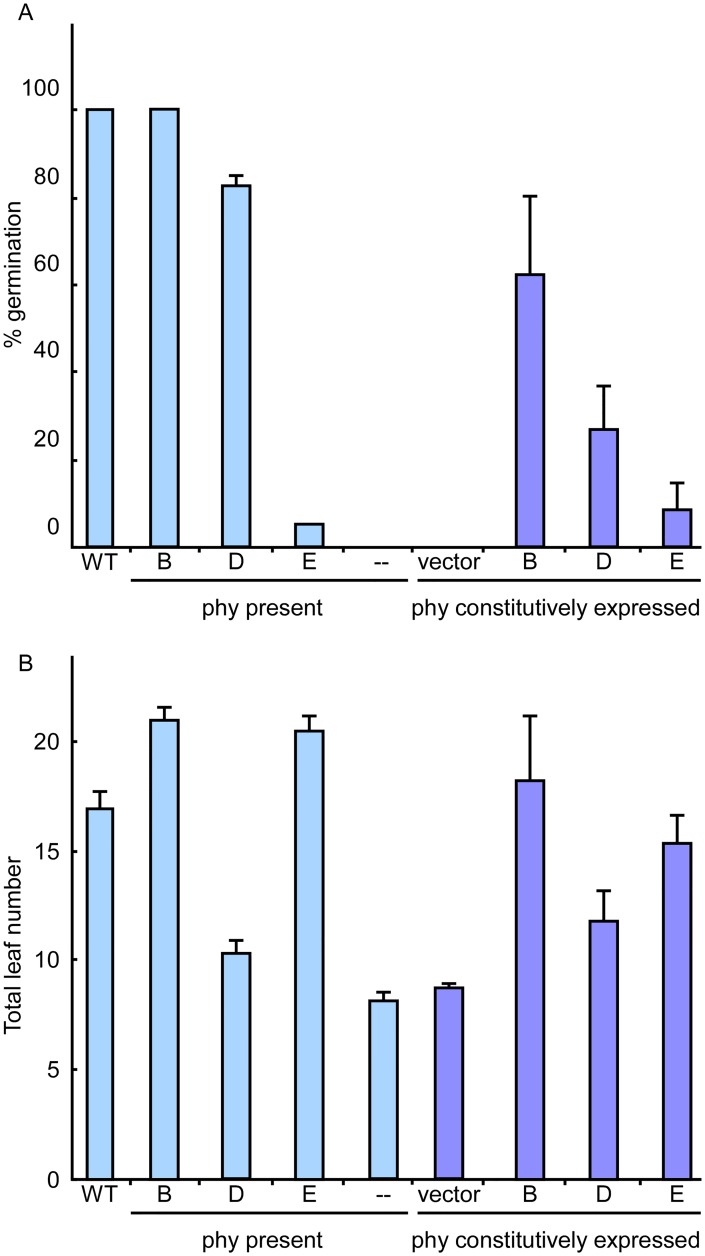
The individual roles of phyB, phyD and phyE depend on protein sequence rather than expression level or pattern. Germination rates (A) and flowering time (B) of independent transgenic lines harboring each phytochrome under the 35S promoter in a background devoid of other phytochromes. Plants harboring the indicated phytochromes were grown under LD conditions at 18°C (B) or under white light at 23°C (A) and total leaf number and germination rates were determined as in Figs 1 and 2. WT, quadruple and quintuple phytochrome mutants were compared to transgenic lines bearing HA tagged versions of phyB, phyD or phyE in the quintuple phytochrome mutant background and the empty vector control lines. Data points represent the mean ±SE of 4 independent transgenic lines for the vector control and 6, 8 and 12 independent lines for the constructs bearing, *35S*:*PHYB*, *35S*:*PHYD* and *35S*:*PHYE* respectively. Quantification of protein levels and the germination and flowering responses of individual lines are shown in (S8 Fig and S2 Table).

PHYD apoprotein was expressed at somewhat lower levels (50% in average), and PHYE to even lower levels (12%), when compared to PHYB expressing lines, which is consistent with previous reports on the overexpression of these photoreceptors [24] (S8 Fig). We used several independent lines, that were also positive for PHY accumulation, to compare the effectiveness of each photoreceptor in the regulation of germination and flowering, in the absence of other phytochromes (Fig 4).

Several 35S:phyD-HA lines restored germination to levels of above 50%, but only weakly delayed flowering, to timing similar to that of the quadruple *phyA phyB phyC phyE* mutant, except for a unique late flowering line (1 out of 8 independent lines). Conversely, 35S:phyE-HA lines did not germinate better than the *phyA phyB phyC phyD* line, except for a single line that expressed phyE to very high levels (1 out of 12 independent lines, S8 Fig), but several of these lines significantly delayed flowering, despite their relative lower levels of apoprotein accumulation (Fig 4 and S2 Table). These results confirm that the roles of phyD and phyE differ due to the nature of the photoreceptors themselves rather than to differences in the expression patterns of their mRNAs.

Lines expressing 35S:phyA-HA did not restore full phyA activity and had only weak effects on flowering time and germination (S7 Fig). phyA is known to accumulate to high levels in etiolated seedlings, about 8-fold more than phyB [31]. In our transgenic lines, phyA-HA accumulated to levels not much higher than phyB-HA in etiolated seedlings (S8 Fig). Hence, these results could be due to differences in expression level compared to that driven by the native phyA promoter [32].

**Fig 5:**
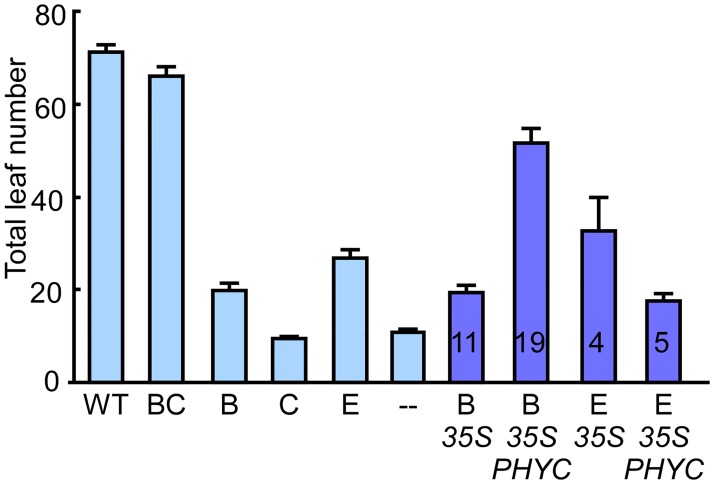
phyB requires phyC to regulate the photoperiodic response. Flowering time of transgenic lines bearing phyC under the 35S promoter in a background containing only phyB or phyE. Plants harboring only the indicated phytochromes were grown under SD conditions at 23°C and flowering time was determined as in Fig 2. The numbers inside each bar represent the number of independent T1 lines used.

Finally, expression of 35S:phyC-HA did not restore germination at all and did not delay flowering significantly on its own, but did delay flowering under SD conditions when transformed into plants bearing only phyB (Fig 5). Further, 35S:phyC-HA also antagonized phyE activity, consistent with previous results (Figs 2 and 5). To account for differences in T-DNA insertion sites, we performed a similar flowering experiment with F1 lines that were each the product of a cross between the 35S:phyC-HA lines (in the quintuple phytochrome mutant background) and lines bearing only phyB (i.e., *phyA phyC phyD phyE* quadruple mutants). Despite being heterozygous for the *PHYB* locus (*PHYB*/*phyB*) and hemizygous for the *35S*:*phyC-HA* insertion, these F1 lines flowered significantly later than the *35S*:*phyC-HA* homozygous lines (in the quintuple mutant background) and the *phyA phyC phyD phyE* quadruple mutants homozygous for *PHYB* (S9 Fig), further confirming the mutual requirement of phyB and phyC for regulating the photoperiodic response.

### phyC modulates the subcellular localization patterns of phyB in different light conditions

Phytochrome heterodimers are known to exist for the phyB/phyC, phyB/phyD, phyB/phyE and phyC/phyD pairs [20, 21]. Phytochrome fusions to fluorescent proteins were instrumental in studies about the dynamics of phytochrome nuclear localization. However, these studies did not distinguish between phytochrome homodimers and heterodimers [22, 33–35]. On the other hand, the interactions among phytochromes evidenced above could be either direct or indirect. To test whether direct interactions were possible and if heterodimers differ in intracellular localization patterns, we examined all possible phytochrome pairs by bimolecular fluorescence complementation (BiFC) analysis (S10 Fig). We fused the C-terminus of each phytochrome to either the N-terminal of the Enhanced Yellow Fluorescence Protein (nEYFP) or the C-terminal (cEYFP). When two molecules of phytochrome interact, the two EYFP halves are close enough to reconstitute the fluorescence activity. In these assays, the complexes mature with time and the equilibria may be displaced, therefore they can not be taken as a measure of binding affinity. On the other hand, we tested the expression of each phytochrome in the *Nicotiana benthamiana* transient system and they were not expressed at similar levels (S8C Fig). Therefore, our assays must be interpreted in a qualitative rather than quantitative manner. We co-expressed each pair of constructs in *Nicotiana benthamiana* leaves and kept the plants in the dark for two days before observing the EYFP fluorescence (S10 Fig). phyC was the only phytochrome that did not yield detectable fluorescence when paired with itself, which is in accordance with reported data that phyC does not form homodimers [21]. By contrast, we detected phyE-phyE interactions. Coupled with the finding that phyE is biologically active in the absence of other phytochromes (Fig 2), this result strongly suggests that phyE forms homodimers. Furthermore, self-interactions were observed for phyA, phyB, and phyD, consistent with these phytochromes forming homodimers [21, 22]. phyA, phyB, phyD and phyE interacted with each other (S10 Fig). The phyA/phyD and the phyA/phyC signal was relatively weaker, but still above background, indicating that these heterodimers may be possible. The interactions of phyC with phyB and with phyD, the interactions of phyD with phyE and with phyB and the interactions of phyE with phyB are consistent with the reported heterodimers [21], whereas the interaction of phyC with phyE, and phyA with phyB and with phyE indicates that the existence of phyC/phyE, phyA/phyB and phyA/phyE heterodimers may also be possible.

**Fig 6:**
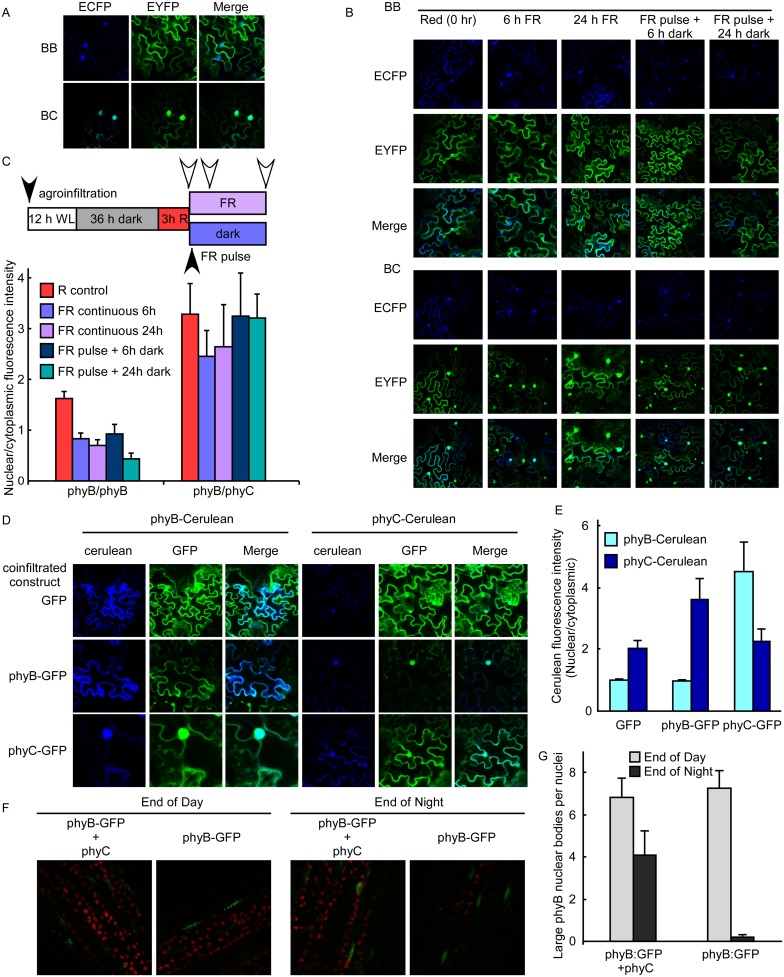
Light conditions differentially affect the intracellular localization patterns of phyB/phyB homodimers and phyB/phyC heterodimers. (**A**) Nuclear/cytoplasmic partitioning of phyB homodimers differs from that of phyB/phyC heterodimers in *Nicotiana benthamiana* transient assays. Each pair or constructs bearing phyC-cEYFP and phyB-nEYFP (BC) or phyB-cEYFP and phyB-nEYFP (BC) were agroinfiltrated in LD-grown *Nicotiana benthamiana* leaves together with ECFP-NLS as a nuclear marker (Blue). The following day, plants were treated with a FR pulse and then grown for two more days in darkness. Leaves were collected under a green safe light and then fixed with formaldehyde in darkness before confocal microscopy examination. (B, C) PhyB/phyC heterodimers are unresponsive to light quality. *Nicotiana benthamiana* leaves from plants grown in LD were agroinfiltrated as in (A), grown for another 12 h in WL, dark adapted for 36 h and then received a R treatment for 3 h. After the R treatment, a set of leaves were collected (R control) and another set of plants received either a pulse of 15 min FR followed by dark or continuous FR, to revert phytochrome to the Pr form. Leaves were collected after either 6 or 24 h after the ending of the R treatment. Leaves were fixed and examined by confocal microscopy. Black arrows in the scheme (C) indicate treatments, while white arrows indicate harvesting points. For quantitative data shown in (C), randomly selected individual cells were used to quantify the fluorescence intensity in three randomly selected areas of the nucleus and the cytoplasm. Nuclear and cytoplasmic intensities were averaged for each cell and then averaged among independent leaves. Data are means ±SE of 6 independent leaves. (D, E) Coexpression of phyB and phyC changes their localization patterns. phyB-Cerulean was coexpressed with either GFP alone as a control, phyB-GFP or phyC-GFP (left set of panels in D) and phyC-Cerulean was coexpressed with either GFP alone as a control, phyB-GFP or phyC-GFP (right set of panels in D). *Nicotiana benthamiana* leaves from plants grown in LD were agroinfiltrated, grown for another 12 h in WL, treated with a pulse of 15 min FR to revert phytochrome to the Pr form and dark adapted for 36 h before confocal microscopy. (E) Images were quantitated as in (C) and data are means ±SE of 6 independent leaves. (F-G) phyC promotes the localization of phyB to large nuclear bodies in Arabidopsis during the night period. Transgenic lines bearing phyB-GFP in the quintuple phytochrome mutant background were crossed to lines either bearing only phyC (*phyA phyB phyD phyE* quadruple mutants) or the quintuple phytochrome mutant as a control. The F1 lines were grown in SD conditions and used to observe the effect of phyC on the localization of phyB-GFP at two time points, 1 h before lights-on (End of Day) and 1 h before lights-off (End of Night). (F) The phytochromes indicated above panels are the only phytochromes present in these F1 lines. Chloroplasts are observed in red, whereas phyB-GFP is observed as green dots within the nuclei (the three left panels) or diffuse green nuclei (the right panel) of Arabidopsis hypocotyl cells. (G) Quantification of large nuclear bodies from confocal images. Data points represent the mean ±SE of 12 nuclei, 4 nuclei from 3 seedlings for each genotype and condition.

Interestingly, fluorescence was mostly localized to the nuclei when phyB was combined with phyC and to some extent when phyD was combined with phyC (S10 Fig). The existence of phyB/phyC heterodimers was recently described [21, 30], but its intracellular localization pattern was never observed. The phyB/phyC heterodimers could have been rapidly transported into the nuclei in response to the light emitted during confocal microscopy. To avoid this, we collected the leaves under a green safe light and fixed the leaves in the dark before confocal microscopy. Again, an important fraction of phyB/phyC fluorescence remained in the nuclei (Fig 6A), lower panel, green nuclei), while in the same conditions most of the phyB/phyB fluorescence was cytoplasmic (Fig 6A, upper panel, green cytoplasm and blue nuclei marked by ECFP fused to the SV40 NLS). This result suggests that phyC alters the nuclei/cytoplasm partitioning of phyB in *Nicotiana benthamiana*. To test this possibility, we compared the rate of reaccumulation of phyB/phyB homodimers and phyB/phyC heterodimers in the cytoplasm in R and FR treated WL-grown plants. After infiltration, plants were grown for 12 h under WL, given a FR pulse and then grown for 36 h in the dark. After the dark period, plants were treated with R for 3 h and analyzed by confocal microscopy (Fig 6B and 6C, R control, time zero). Both phyB/phyB homodimers and phyB/phyC heterodimers were localized to both cytoplasm and nuclei. However, after treatments with FR, a substantial amount of phyB/phyC heterodimers remained in the nuclei (green nuclei), whereas most of the phyB/phyB homodimers were cytoplasmic (green cytoplasm and blue nuclei, Fig 6B and 6C). To further test these localization patterns with a BiFC independent assay, we evaluated the localization of phyC and phyB when coexpressed with each other (Fig 6D and 6E). For this purpose we used GFP and Cerulean fusions of both phyB and phyC. After agroinfiltration with these constructs, plants were grown for 12 h under WL, given a FR pulse and then grown for 36 h in the dark. After the dark period, leaves were collected under a safe green light, fixed and analyzed by confocal microscopy. Once again we observed that phyB was mostly cytoplasmic when expressed alone, whereas it was mostly nuclear when coexpressed with phyC. Conversely, the phyC pattern was mostly nuclear when coexpressed with phyB. Contrary to BiFC data (S10 Fig), phyC was detected when expressed alone (Fig 6D), suggesting that it was stabilized by association with endogenous tobacco phytochromes. Interestingly, phyC was also more nuclear localized than phyB. These results suggest that phyC forces phyB to localize to the nucleus, after periods of darkness and in a light-quality independent manner. However, in Arabidopsis nuclei, phyB remains nuclear under prolonged periods of darkness, low quality or low irradiance, but changes its pattern of localization in nuclear bodies, from large to small nuclear bodies [36, 37]. To address the behavior of phyB in the presence of phyC in Arabidopsis, we generated phyB-GFP lines either in the quintuple phytochrome mutant background or in a background having only phyC, by crossing *phyB-GFP phyA phyB phyC phyD phyE* lines with either the quintuple *phyA phyB phyC phyD phyE* mutant or the *phyA phyB phyD phyE* quadruple mutant bearing only phyC. The F1 lines were grown in SD conditions in which we observed effects of phyC and phyB on hypocotyl elongation (S6B Fig) and flowering (Fig 2B). We observed the pattern of phyB localization at two time points, during the last hour of a SD and during the last hour of a long night (Fig 6F and 6G). phyB-GFP was localized to large nuclear bodies after the light period regardless the presence of phyC. However, after 15 h in the dark, phyB-GFP remained in large nuclear bodies in the presence of phyC, whereas in the absence of phyC, phyB-GFP showed a diffused pattern within the nuclei (Fig 6F and 6G). It was recently shown that phytochrome nuclear bodies are required to inhibit hypocotyl elongation during a prolonged dark period [37]. In the light of these findings, our results strongly suggest that phyC is important to maintain phyB in these active nuclear bodies. Besides the pattern of nuclear localization, phyC could affect total nuclear phyB. We measured phyB and phyC levels in nuclear extracts of plants bearing only phyB, only phyC or both (S11A and S11B Fig) during the last hour of a SD or the last hour of the long night period. As expected, phyC did not accumulate in the absence of phyB (S11B Fig), but phyB nuclear levels were higher in the presence of phyC (S11A Fig), suggesting that phyB/phyC heterodimers may be more stable within the nuclei than phyB homodimers. To further test if phyC could increase the activity of phyB at the end of the night phase, we measured the expression of genes that respond to either light quality or dawn cues during the dark to light transition (S11C–S11F Fig), a condition where phyC promotes de accumulation of phyB in large nuclear bodies (Fig 6F and 6G). The mRNA levels of *PIF3-LIKE 1* (*PIL1*) and *ARABIDOPSIS THALIANA HOMEOBOX PROTEIN 2* (*ATHB2*) were repressed by the photoreceptors phyB and phyC to a lower level than phyB alone, even during the last hour of the dark period (S11C and S11D Fig) [37]. The morning-expressed clock genes *NIGHT LIGHT-INDUCIBLE AND CLOCK-REGULATED1* (*LNK1*) and *CIRCADIAN CLOCK ASSOCIATED 1* (*CCA1*) [38, 39] showed the strongest response to light when both phyB and phyC were present (S11E and S11F Fig). Interestingly, the phase of *CCA1* expression was delayed in plants bearing phyB and phyC compared to plants bearing phyB alone. This could be a mechanism underlying the sensitivity of plants bearing phyB and phyC to photoperiod. Taken together, these gene expression studies show that the coordinated action of phyB and phyC is observed when the nuclear phyB/phyC heterodimers are expected to be relatively more abundant in large nuclear bodies, at the dark to light transition after a long night period.

## Discussion

During the past 25 years or so, phytochrome mutants have been extensively used to study the roles of phytochromes. phyB and phyA have emerged as the most important phytochromes [1, 2, 18, 29], whereas the roles of phyD, phyE, and phyC have been assumed to be minor and redundant with phyB [6, 11, 12, 14, 15, 22, 24, 40, 41]. However, our knowledge about the capacity of individual phytochromes to elicit specific responses and the interactions among them is still incomplete.

### The individual roles of each phytochrome and their interactions

When present alone, phyC is barely active, but causes a slight decrease in GA sensitivity during germination (S3B Fig), a slight inhibition of hypocotyl elongation under R, blue and white-light (Fig 3; S6 and S12A Figs), and an increase in hook opening (S12B Fig), consistent with previous reports [14, 40, 42]. This low residual activity of phyC is also consistent with the lack of detectable phyC homodimerization [21] (S10 Fig) and the low accumulation of phyC in the absence of phyB (S2 Fig) [20, 27]. However, *Triticum aestivum* (wheat) phyC homodimerizes in Arabidopsis and elicits photomorphogenic responses [43], and forcing homodimerization of phyC triggers photomorphogenic responses in Arabidopsis [22]. Hence, it is possible that the residual effects of phyC may be due to very low levels of phyC homodimers.

It is unclear whether phyE is active independently of the other phytochromes and whether it forms homodimers. Clack et al. did not detect phyE homodimers [21], but more recently, expressed phyE-GFP in transgenic plants was shown to form homodimers [22]. However, native phyE was not tested in a background devoid of all other phytochromes. Our results suggest that native phyE forms homodimers, as phyE is biologically active in the absence of other phytochromes (Figs 1, 2 and 4; S6 and S7 Figs) and it interacts with itself in a BiFC assay (S10 Fig). Nevertheless, it cannot be ruled out that the lack of homodimerization of phyE reported previously may be due to natural phyE variants, or to the use of different accessions [21, 22].

**Fig 7:**
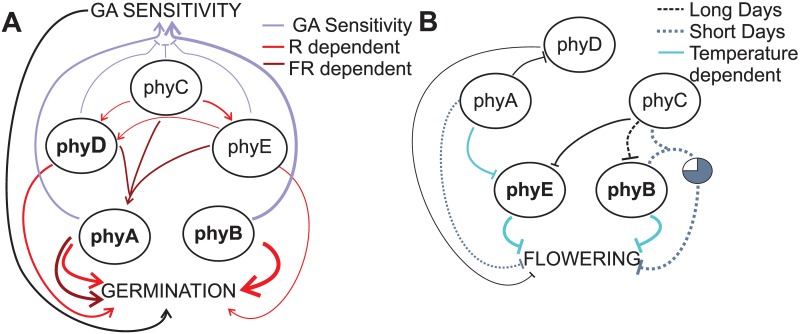
Model of the phytochrome network. Summary of the roles of phytochromes and the interactions between them during germination (A) and flowering (B). Positive interactions are depicted by arrows and negative interactions by lines. The thickness of each line indicates the strength of the action.

Interestingly, we found that phyE repressed flowering, even to a greater extent than did phyB (Fig 2). This repression was highly dependent on ambient temperature under LD conditions, accounting for the temperature-dependent flowering of *phyB* mutants [13, 41]. Thus, phyE might compensate for the lack of phyB at low temperatures. Conversely, the individual effects of phyE on germination and hypocotyl elongation were subtle (Figs 1, 3 and 4; S1, S3 and S7B Figs; S2 Table), but interactions with other phytochromes emerged (see below, Fig 7).

In contrast to phyE, phyD was more efficient to promote germination (Figs 1 and 4; S1 and S7B Figs; S2 Table), but only weakly repressed flowering (Figs 2 and 4; S7A Fig; S2 Table). Hence, phyD and phyE, which were previously believed to work mostly in a redundant fashion [6, 11, 12, 14, 15, 22, 24, 40, 41], have distinct roles. At this point it is unclear how this may occur. It might imply different capacity of phyD and phyE to interact with downstream signaling components. On the other hand, even if our data does not support that general changes in phytochrome stability may explain the differences between phyD and phyE, we cannot rule out that specific mechanisms to regulate the level of each phytochrome in a tissue specific manner may exist and could account for the opposite efficiencies with which phyD and phyE promote germination and repress flowering.

After phyB, phyA was the most important promoter of germination and GA sensitivity, and these effects were more evident under FR (Fig 1; S3 Fig). These findings are consistent with the established roles of phyA [18]. However, rather than behaving as a flowering promoter [28], phyA, when present in isolation, turned out to be a weak flowering repressor, strongly suggesting that phyA promotes flowering in an indirect manner, by modulating the signaling pathway transduced by phyE and, to a lesser extent, phyD (Figs 2 and 7). Further, phyA was the only phytochrome to confer some degree of photoperiod sensitivity on its own, showing a stronger repressive role in SD conditions (Fig 2B).

As expected, phyB was the phytochrome that had the greatest stimulatory effect on germination and GA sensitivity (Fig 1; S1, S3 and S7B Figs) and the greatest inhibitory effect on hypocotyl elongation (Fig 3). Despite the widely accepted role for phyB in the photoperiodic flowering response [29], phyB did not confer photoperiod sensitivity on its own, showing that phyB is necessary but not sufficient for the photoperiodic response (Fig 2B).

In our analysis of binary interactions, we detected both positive and negative interactions (Fig 7). Whereas phyC, phyD, and phyE acted synergistically to promote germination in response to light (Fig 1; S1 Fig), they antagonized both phyA and phyB in terms of GA sensitivity (Fig 2; S1 Table). As GA promotes germination but antagonizes phytochromes in the control of hypocotyl elongation [1], this mechanism may allow seedlings to regain phytochrome control of hypocotyl elongation (seedling emergence) after germination. Interestingly, phyC, phyD, and phyE also acted synergistically with phyA to inhibit hypocotyl elongation under blue light (S6A Fig). This synergistic effect, similar to that observed for FR-promoted germination (Fig 1 and S3 Fig), may be due to higher protein levels of phyA in the presence of any of the three phytochromes, phyC, phyD or phyE (Fig 7 and S2 Fig).

Our results also highlight the importance of the antagonistic effects of phytochromes in achieving proper flowering time. Plants bearing only phyB or phyE flowered significantly later than did WT plants under LD conditions, underscoring the importance of phyA and phyC antagonism mainly on phyE and phyB, respectively (Figs 2 and 7).

The interactions among phytochromes shown here could be due, at least in part, to the formation of heterodimers. Heterodimers of phyB/phyC, phyB/phyD, phyB/phyE, and phyD/phyE, and, to a lesser extent, phyD/phyC were previously shown to exist [20, 21]. However, heterodimers containing phyA and phyC/phyE heterodimers were not found. In this study, we detected interactions between phyA and all phytochromes (including a very weak signal for phyA/phyC) and also phyC/phyD and phyC/phyE interactions by using BiFC (S10 Fig). These results must be taken with caution as agroinfiltration in tobacco leaves may lead to high expression levels (S8D Fig). Nevertheless, these results raise the possibility that the pair wise interactions observed may be due, at least in part, to direct protein-protein interactions among the phytochrome members and suggest that the phytochrome signaling network is more complex than previously thought.

### phyB and phyC coaction is essential and sufficient for photoperiodism

The role of phyB in the photoperiodic response has been extensively reported in diverse species [29, 44, 45]. However, although phyB can repress flowering on its own, it requires the presence of phyC to confer photoperiod responsiveness (Fig 2 and S5 Fig). On the other hand, phyC requires phyB, consistent with previous reports in both rice and Arabidopsis [15, 30, 40]. The strict requirement for the activity of both phyB and phyC could indicate that components downstream of the photoreceptors interact or that the activity of phyB/phyC heterodimers differs from that of the individual photoreceptors. Our data favor the second possibility. First, the existence of phyB/phyC heterodimers was reported previously in both Arabidopsis and rice [21, 30] and we have shown here that phyB and phyC interact in vivo (Fig 6 and S10 Fig). Second, we showed that the phyB/phyC heterodimers are consistently localized to the nucleus in transient assays in *Nicotiana benthamiana*, even after prolonged dark periods or under low red to far-red light ratios, suggesting an emerging property of the phyB-phyC system (Fig 6 and S10 Fig). Third, neither phyB nor phyC conferred even a subtle photoperiodic response on their own; this is unlikely if phyB and phyC coaction is due to the interaction of downstream components of phyB and phyC. Interestingly, phyB repressed hypocotyl elongation on its own in response to R (Fig 3) and repressed flowering under both SD and LD conditions (Fig 2), but only in the presence of phyC was phyB able to restore photoperiodism.

Recent reports strengthen the idea that phyB in large nuclear bodies is active and necessary to trigger downstream processes during the night period [35–37]. We show here that phyC promotes the localization of phyB to large nuclear bodies after a long night period (Fig 6F and 6G), suggesting that phyB/phyC heterodimers are active for longer periods of darkness. Hence, the extended activity phyB/phyC heterodimers in the night and its availability early in the dark to light transition might be important to repress flowering and hypocotyl elongation specifically under SD conditions.

Two interesting previous observations may be explained in light of our results. It was reported that *PHYB* antisense lines with a ~75% reduction in phyB display longer hypocotyls, as do *phyB* mutants, but normal flowering time [46]. Similarly, the *phyB-28* allele, which lacks most of the HKRD domain, has long hypocotyls but an almost normal flowering time [47]. These observations can be explained if, under low phyB levels, phyB is found mostly as a heterodimer with phyC and hence retains normal photoperiodic responses with respect to flowering, while impairing normal hypocotyl responses that are mostly due to phyB/phyB homodimers. Similarly, the effects of the *phyB-28* allele could be explained if this mutation either affected phyB levels (which may not be the case) or if phyB/phyC heterodimers were more abundant than phyB/phyB homodimers.

We show here that phyB alone is sufficient to confer full hypocotyl and germination responses to R and to repress flowering, which is also consistent with phyB alone being sufficient to confer a response to light quality (response to R/FR ratios) [42]. However, it has not been easy to dissect the role of phyB in the flowering response to light quality from its role in photoperiodic flowering [28, 29, 48]. Our data support the notion that phyB/phyB homodimers are involved in the responses to light quality, whereas the phyB/phyC heterodimers are involved in the photoperiodic response. The role of phyC in photoperiodism may be widely conserved. In population studies, strong phyC alleles were found to be more abundant at higher latitudes [49], which could indicate that these alleles have an increased sensitivity to photoperiod. In wheat, *Brachypodium distachyon*, and *Hordeum vulgare* (barley), phyC promotes flowering more effectively in LD conditions [43, 50, 51]. These results highlight the importance of phyC in photoperiodic responses in diverse habitats and species and are consistent with our finding that phyC is essential for the photoperiodic response. It would be interesting to study if phyC also promotes flowering under LD conditions by antagonizing phyB in wheat and related grasses and to establish the possible involvement of phyB/phyC heterodimers in the photoperiodic response of these species.

### Novel interactions between temperature and the phytochrome system

An interesting aspect of phytochromes is that their effects are altered by temperature (Fig 2A). An absence of phytochromes resulted in very low temperature sensitivity under LD conditions. However, phyE and phyB repressed flowering more efficiently as the temperature decreased, indicating that cross-talk exists between the phytochrome and temperature signaling pathways (Fig 2A). The specific effect of phyA on temperature-dependent phyE signaling, but not on phyB signaling, strongly suggests that there are differences in the signaling pathway downstream phyB and phyE (Fig 2A). How phyE regulates flowering is still unknown, but these results raise the possibility that a CONSTANS (CO)-independent mechanism that differs from the phyB-mediated effect on CO stability may function downstream of phyE [29]. We think of two possible mechanisms to explain how phyB/phyC heterodimers might contribute to photoperiod detection. One possibility is that the effects of phyB on CO stability [29] may be due indeed to phyB/phyC heterodimers. A second possibility is supported by the role of phyB in regulating the phase of the circadian clock [52] and also supported by our gene expression data (S11 Fig): phyB/phyC heterodimers might affect the phase of clock and flowering time genes and hence, photoperiod detection.

Flowering was reported to be insensitive to the photoperiod in the absence of phytochromes [10], and we found similar results when plants were grown at 24°C. However, we also found that photoperiod responsiveness was restored at low temperatures (Fig 2B). Together, these results suggest the existence of phytochrome-dependent and -independent mechanisms that regulate the flowering response to temperature, consistent with previous genetic evidence [53]. PIF4 [54] and two transcription factors that form heterodimers, SHORT VEGETATIVE PHASE (SVP) and FLOWERING LOCUS M (FLM/MAF1), regulate flowering in response to ambient temperature [55, 56]. Further experimentation is needed to determine if the PIF4 and SVP/FLM pathways correspond to phytochrome-dependent and -independent pathways.

## Materials and Methods

### Plant material and growth conditions

*phyA-211*, *phyB-9*, *phyC-2*, *phyD-201*, and *phyE-201* alleles are in the Columbia background [4, 15, 42]. Segregating populations were genotyped as previously described [42] to identify triple and quadruple mutants.

For experiments with seedlings, sterilized seeds were suspended in 100 μM GA_4+7_ (Duchefa Biochemie, Haarlem, The Netherlands), stratified for 3 days at 4°C, and then pipetted onto plates of Murashige Skoog Salts media and 0.8% Plant Agar (Duchefa Biochemie). Light treatments were performed in dedicated growth chambers (Model I30BLL, Percival Scientific, Perry, IA, U.S.A.). For red and far-red light, light-emitting diodes were used. For the hypocotyl measurement assays, 15 seeds were plated per replicate, and the average height of the 10 tallest seedlings was recorded per replicate. In the germination assays, sterilized seeds were directly plated on MS salts plates (0.8% agar) and given a post-imbibition saturating 5-min FR pulse to revert seed phytochrome to the Pr form. Then, the seeds were stratified in darkness for 3 days at 4°C. After stratification, seeds were incubated at 23°C for 6 days under the indicated light regimes, before counting the germinated seeds (i.e., radicle emergence). Each pool of seeds used in the germination assays was collected from plants grown side by side under the same conditions. This process was repeated several times and pools of seeds grown under the same conditions, but at different times, were collected.

### Generation of transgenic plants

The phytochrome cDNAs were obtained by retrotranscription from Col-0 RNA, and cloned into the pCHF5 plasmid fused to the C-terminus HA tag (S3 Table). Single locus insertion lines from the T3 generation were selected for each experiment. (See also Supporting Information.)

### Bimolecular fluorescence complementation assay

*Agrobacterium tumefaciens* (GV3301) containing the pCardo1-C-nEYFP or pCardo1-C-cEYFP vector (harboring each phytochrome tagged with nEYFP or cEYFP, respectively), the pBIN19-35S-P19 vector (containing the P19 suppressor of silencing), and the pCardo2.1-ECFP-NLS vector (containing the nuclear marker ECFP-NLS) were co-infiltrated into the leaves of *Nicotiana benthamiana* plants grown under LD at 23°C essentially as described [57] with some modifications. After infiltration, plants were grown in the same LD conditions for 12 h, up to the end of the photoperiod, and then treated with FR pulses, R pulses or darkness, as indicated in each figure legend. The confocal images were taken using a Zeiss LSM 710 microscope. EYFP was excited at 514 nm and observed at 520–539 nm, whereas ECFP was excited at 458 nm and observed at 466–480 nm.

### Nuclei enrichment and immunoblots of nuclei extracts

600 mg of tissue were frozen in liquid nitrogen and gently grinded in a mortar. Then, the nuclei extraction was performed as an simplified version of [58] without the Percoll gradient. The nuclei was pelleted and then washed twice to enrich in the nuclei fraction. Equal volumes of each nuclei preparation were used for immunoblots, and phytochromes quantitated relative to H3.

### Accession numbers

The sequence of genes used in this study can be found in the GenBank/EMBL or the Arabidopsis Genome Initiative databases under the following accession numbers: AT1G09570 (*PHYA*), AT2G18790 (*PHYB*), AT5G35840 (*PHYC*), AT4G16250 (*PHYD*), AT4G18130 (*PHYE*), AT4G16780 (*ATHB2*), AT2G46970 (*PIL1*), AT2G46830 (*CCA1*). AT5G64170 (*LNK1*).

## Supporting Information

S1 Fig The regulation of germination by single phytochrome photoreceptors and their synergistic interactions under white light. Seeds of the indicated genotypes were stratified as described in Materials and Methods, and then incubated for 6 days under continuous WL (50 μmol m^-2^ s^-1^) at 23°C before the germinated seeds (radicle visible) were counted. Data are averages ± SE of 16 independent plates with 20 seeds each and 4 independent seed pools (collected from independently grown plants).
(PDF)

S2 Fig Phytochrome protein levels in triple and quadruple phytochrome mutants. Plants bearing only one or two phytochromes were used to analyze how each phytochrome was affected by the other family members. Protein levels of each phytochrome apoprotein were determined by immunoblot, using specific monoclonal antibodies [27]. Seedlings of each genotype were grown for seven days in either continuous white light (60 μmol m^-2^ s^-1^) or continuous darkness. Total protein in extracts was quantified for equal protein loading in each lane (25μg for phyA and phyB and -100μg for phyC, phyD and phyE). Letters above each panel indicate the phytochromes present, whereas the arrows indicate the phytochromes detected by monoclonal antibodies. Below each panel, the numbers indicate relative band intensities within each panel.
(PDF)

S3 Fig Antagonistic interactions between the phytochromes affect GA sensitivity during germination. (A and B) Seeds harboring the indicated phytochromes (the corresponding genotypes are shown on Fig 1) were plated on MS salts agar plates containing 100 μM Paclobutrazol and 0.1 μM GA (A), or 100 μM Paclobutrazol and 10 μM GA (B). After stratification, the seeds were incubated for 6 days under white light (50 μmol m^-2^ s^-1^) at 23°C before the germinated seeds were counted. Data are averages ± SE of 20 independent plates with 16 seeds each and 4 independent seed pools. The complete dataset is presented in S1 Table. (C) Seeds harboring the indicated phytochromes on the abscissas were plated on MS salts agar plates containing 100 μM Paclobutrazol and 1 μM GA. Germination rates were determined as above after treatments with either continuous R or FR. Data are averages ± SE of 8 independent plates with 20 seeds each and 4 independent seed pools (collected from independently grown plants).
(PDF)

S4 Fig Days to flowering in triple and quadruple phytochrome mutants. Plants bearing the indicated phytochromes were grown under long days (LD, 16 h light/8 h dark) (A) or LD and short days (SD, 8 h light/16 h dark) (B), at temperatures ranging from 18 to 24°C. LD data in (B) are the same as in (A) and included for the purpose of direct comparison. Days to flowering were recorded at the time of appearance of the first open flower. Data points represent the mean ±SE of at least 10 plants for each genotype and condition.
(PDF)

S5 Fig The photoperiodic pathway requires phyB and phyC at 24°C. The photoperiodic effect was obtained from data on Fig 2 as the difference between flowering in SD minus flowering in LD for each temperature ±SE.
(PDF)

S6 Fig Individual phytochrome effects on the hypocotyl response under Blue-light and White-light photoperiods. Plants bearing the indicated phytochromes were stratified for 3 days at 4°C in the dark in a solution of 100 μM GA_4+7_ and then plated on MS salts agar plates and incubated at 23°C either under continuous Blue-light (20 μmol m^-2^ s^-1^) (A) under White-light photoperiods (50 μmol m^-2^ s^-1^) (B) or kept in darkness (control) for 5 days. Hypocotyls were measured and the values are given relative to the corresponding dark control in each independent experiment. Data are averages ± SE of four independent plates.
(PDF)

S7 Fig The effect of constitutive expression of phytochromes in the quintuple phytochrome mutant background. Flowering time (A) and germination rates (B) of independent transgenic lines harboring each phytochrome under the 35S promoter in a background devoid of other phytochromes. Plants harboring the indicated phytochromes were grown under LD conditions at 18°C (A) or under white light at 23°C (B) and total leaf number and germination rates were determined as in Figs 1 and 2. Box plots represent data from 4 transgenic independent lines for the vector control and 13, 8, 14, 10, and 14 independent lines for the constructs bearing *35S*:*PHYA*, *35S*:*PHYB*, *35S*:*PHYC*, *35S*:*PHYD*, and *35S*:*PHYE*, respectively.
(PDF)

S8 Fig PHY::HA apoprotein accumulation in transgenic *phyA phyB phyC phyD phyE* mutants. (A, B) Independent transgenic lines for the constructs *35S*:*PHYA*::*HA*, *35S*:*PHYB*::*HA*, *35S*:*PHYC*::*HA*, *35S*:*PHYD*::*HA*, and *35S*:*PHYE*::*HA* were grown in the dark for seven days. After grinding, total protein was determined in supernatants and 50 μg of each sample were subjected to SDS-PAGE, and immunoblots detected with anti-HA monoclonal antibodies (Roche 3F10, 2013819). Quantification of bands in (B) are shown in S2 Table. (C, D) Transient expression of constructs *35S*:*PHYA*::*HA*, *35S*:*PHYB*::*HA*, *35S*:*PHYC*::*HA*, *35S*:*PHYD*::*HA*, and *35S*:*PHYE*::*HA* in tobacco leaves by agroinfiltration and its comparison to selected Arabidopsis transgenic lines from (A) and (B) is shown in (D). Tobacco plants were kept in the dark for two day before harvest and tobacco extracts are indicated by “T” over each lane.
(PDF)

S9 Fig phyC is required for the photoperiodic response regulated by phyB. Independent transgenic lines bearing phyC under the 35S promoter were crossed with quadruple phytochrome mutants bearing only phyB. F1 lines were grown under SD at 23°C and the total leaf number was determined as in Fig 2. Data points represent the mean ±SE of at least 12 plants for each genotype.
(PDF)

S10 Fig BiFC assays between all possible pairs of phytochromes. Each possible pair of phytochromes was transiently co-expressed in *Nicotiana benthamiana* leaves as a fusion to either the N-terminal portion of Enhanced Yellow Fluorescence Protein (nEYFP, indicated on the left) or the C-terminal portion of EYFP (cEYFP, indicated above the panels). Plants remained in the dark for two days before confocal microscopy. Negative controls, nEYFP alone paired with phytochrome-cEYFP are shown in the bottom panels.
(PDF)

S11 Fig Coordinated action of phyB and phyC during the dark to light transition. (A-B) Photoreceptor levels in the nucleus during at the end of day and end of night periods. Seedlings of the genotypes bearing only the phytochromes indicated above each lane and the WT control bearing all five phytochromes were grown under SD conditions for 7 days and harvested 1 h before lights-on and 8 h later, 1 h before lights-off. After nuclei enrichment (See Materials and Methods), proteins were detected by immunoblots using either anti phyB (A) or anti phyC (B) monoclonal antibodies. Each band was quantified relative to Histone 3 (bottom panels). Below each lane, the numbers indicate relative quantities of each of PHYB (A) or PHYC (B) apoproteins. **(C-F) Coordinated action of phyB and phyC to regulate gene expression during the dark to light transition**. Seven day-old seedlings of the indicated genotypes (labels indicate the phytochromes present) were grown in SD (8h light/16 h dark) for seven days and harvested during the dark to light transition as indicated. Transcription levels of shade induced genes *ATHB2* (C) and *PIL1* (D), and morning-expressed clock genes *CCA1* (F) and *LNK1* (G) were determined by quantitative real-time PCR, relative to *UBQ10* controls. The Error bars represent SE of three biological replicates, and * indicate P < 0.05, by one-way-ANOVA and Tukey contrasts between B and BC.
(PDF)

S12 Fig Subtle effects of phyC in the absence of other phytochromes. Subtle effects of phyC in the absence of other phytochromes. Phytochromes present are shown. All transgenic lines were generated in the *phyA phyB phyC phyD phyE* background and the construction utilized is indicated. Seeds were stratified for 3 days at 4°C in the dark in a solution of 100 μM GA_4+7_, plated on MS agar plates, and incubated at 23°C either under continuous red (R) light (20 μmol m^-2^ s^-1^) or kept in darkness (control) for 5 days. (A) Hypocotyls were measured as indicated in Materials and Methods and the values obtained under R are presented relative to the corresponding dark control in each independent experiment. Data are averages ± SE of four independent plates. (B) Apical hook opening was determined as the angle between the apical hook and the hypocotyl, taking the average data value for each genotype in each plate as the experimental unit. Data are averages ± SE of four independent plates.
(PDF)

S1 Table GA sensitivity of phytochrome triple and quadruple mutants compared to the quintuple phytochrome mutant. (PDF)

S2 Table Germination and flowering time of transgenic quintuple phytochrome mutant plants constitutively expressing phyB, phyD or phyE. The relative quantification of each band corresponding to tagged versions of phytochrome is included (data obtained from S8B Fig).
(PDF)

S3 Table Primers used in this study. (PDF)

S1 Text (DOC)
