# Supplementary figures and images for: Bottom-up Assembly of the Phytochrome Network
Source: PLoS Genet. 2016 Nov 7;12(11):e1006413. doi: 10.1371/journal.pgen.1006413 (PMC5098793; doi:10.1371/journal.pgen.1006413)

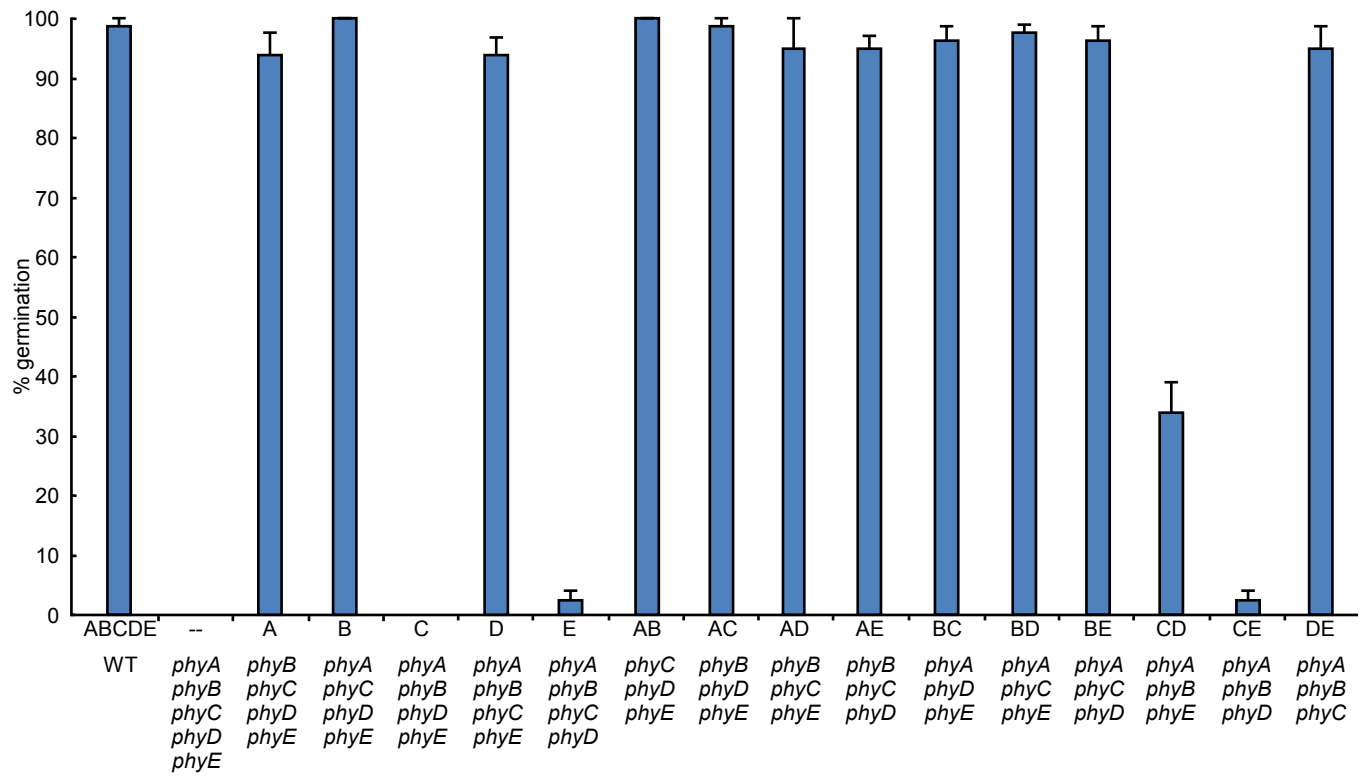

Supplement: S1 Fig — Seeds of the indicated genotypes were stratified as described in Materials and Methods, and then incubated for 6 days under continuous WL (50 μmol m-2 s-1) at 23°C before the germinated seeds (radicle visible) were counted. Data are averages ± SE of 16 independent plates with 20 seeds each and 4 independent seed pools (collected from independently grown plants). (PDF) [file pgen.1006413.s001.pdf]

PHYA/Light

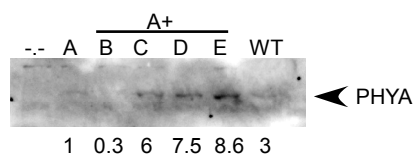

PHYB/Light

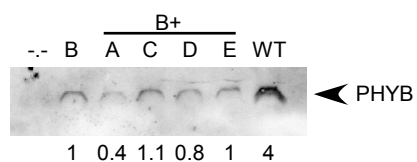

PHYC/Light

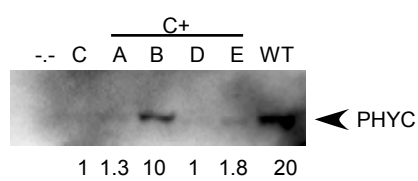

PHYD/Light

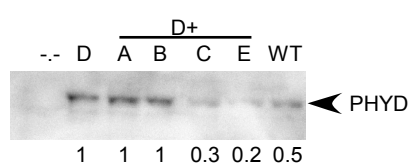

PHYE/Light

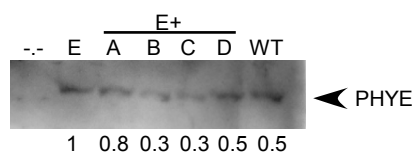

PHYA/Dark

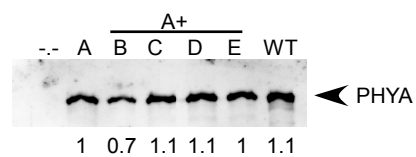

PHYB/Dark

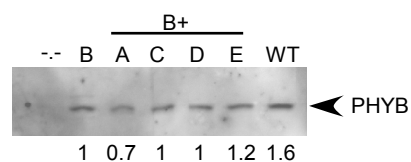

PHYC/Dark

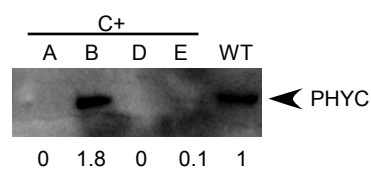

PHYD/Dark

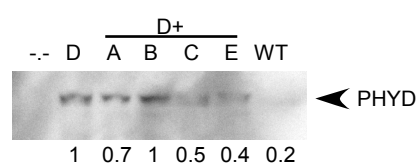

Supplement: S2 Fig — Plants bearing only one or two phytochromes were used to analyze how each phytochrome was affected by the other family members. Protein levels of each phytochrome apoprotein were determined by immunoblot, using specific monoclonal antibodies [27]. Seedlings of each genotype were grown for seven days in either continuous white light (60 μmol m-2 s-1) or continuous darkness. Total protein in extracts was quantified for equal protein loading in each lane (25μg for phyA and phyB and -100μg for phyC, phyD and phyE). Letters above each panel indicate the phytochromes present, whereas the arrows indicate the phytochromes detected by monoclonal antibodies. Below each panel, the numbers indicate relative band intensities within each panel. (PDF) [file pgen.1006413.s002.pdf]

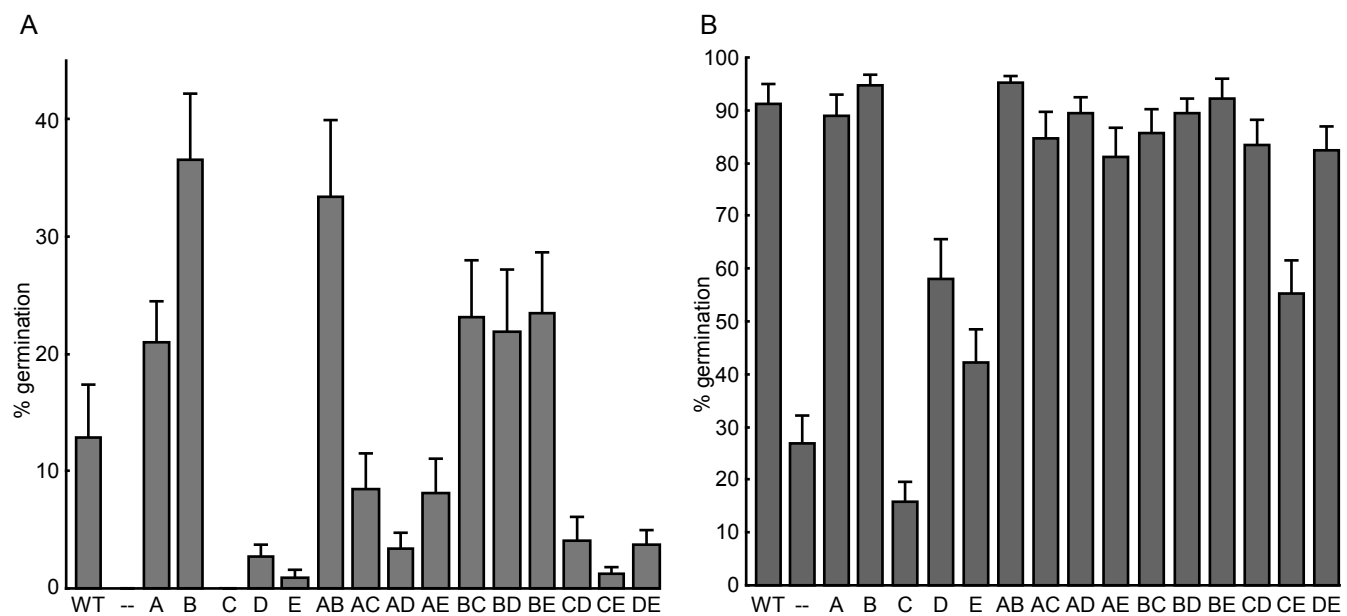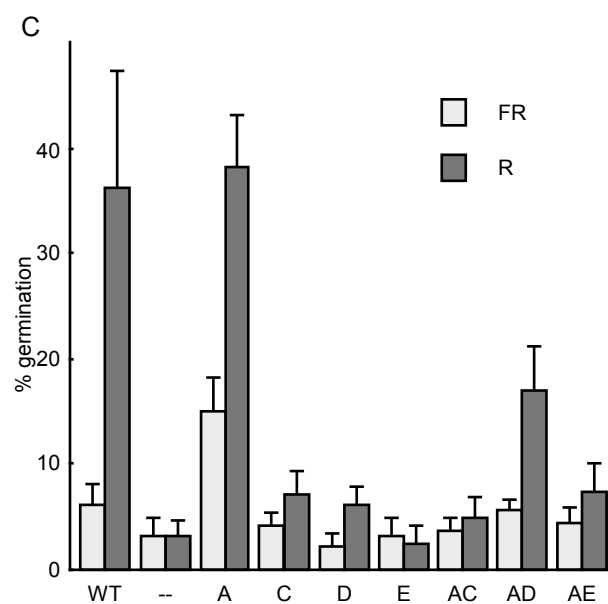

Supplement: S3 Fig — (A and B) Seeds harboring the indicated phytochromes (the corresponding genotypes are shown on Fig 1) were plated on MS salts agar plates containing 100 μM Paclobutrazol and 0.1 μM GA (A), or 100 μM Paclobutrazol and 10 μM GA (B). After stratification, the seeds were incubated for 6 days under white light (50 μmol m-2 s-1) at 23°C before the germinated seeds were counted. Data are averages ± SE of 20 independent plates with 16 seeds each and 4 independent seed pools. The complete dataset is presented in S1 Table. (C) Seeds harboring the indicated phytochromes on the abscissas were plated on MS salts agar plates containing 100 μM Paclobutrazol and 1 μM GA. Germination rates were determined as above after treatments with either continuous R or FR. Data are averages ± SE of 8 independent plates with 20 seeds each and 4 independent seed pools (collected from independently grown plants). (PDF) [file pgen.1006413.s003.pdf]

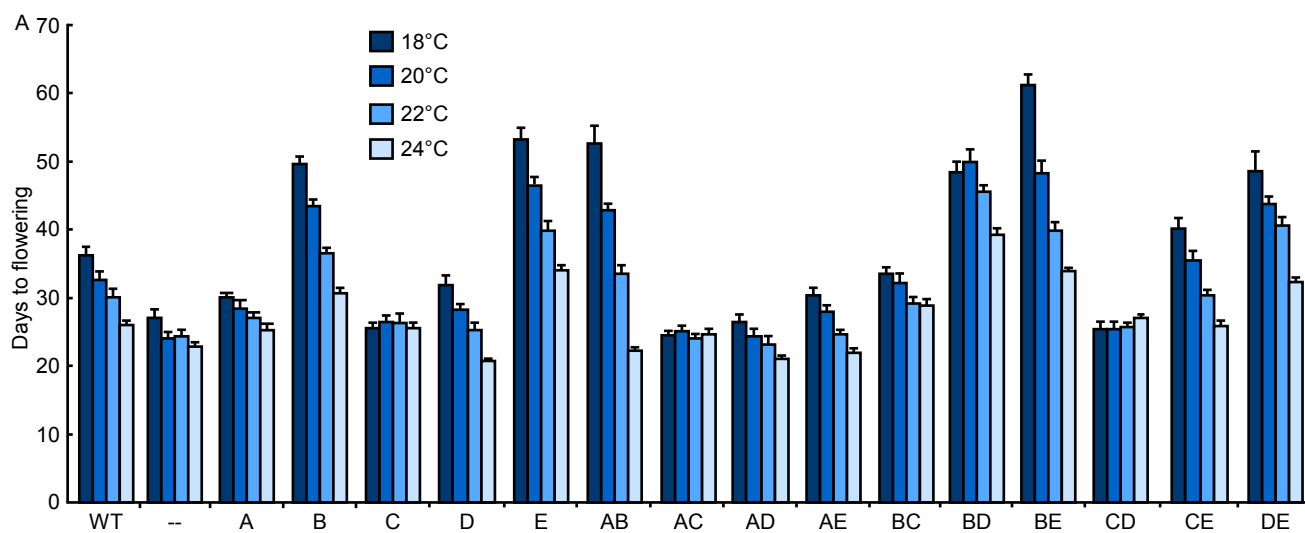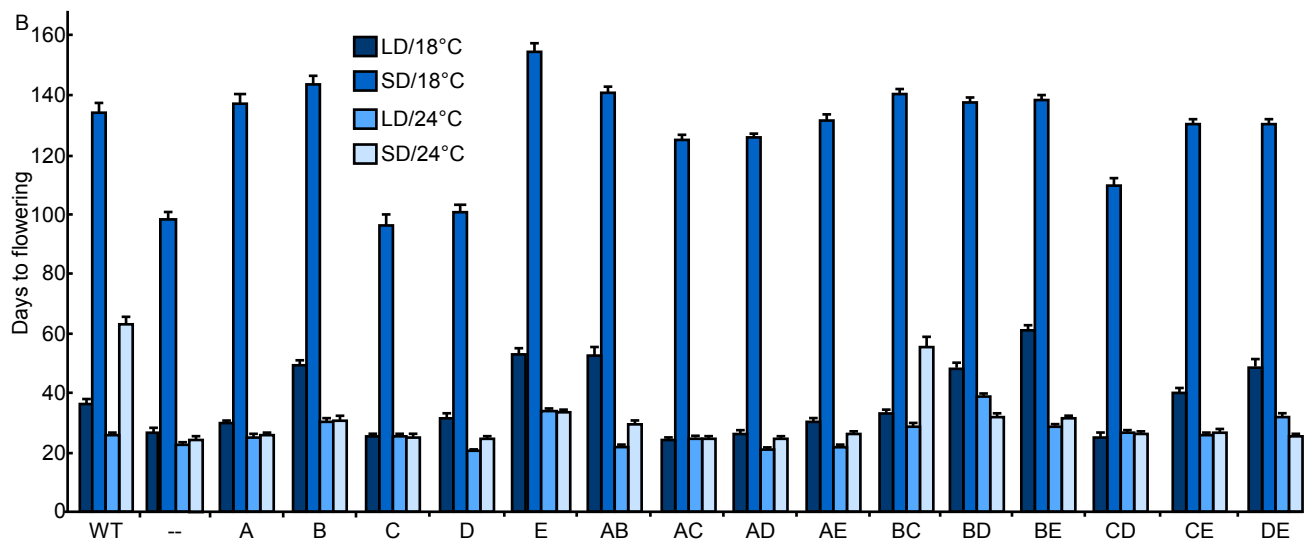

Supplement: S4 Fig — Plants bearing the indicated phytochromes were grown under long days (LD, 16 h light/8 h dark) (A) or LD and short days (SD, 8 h light/16 h dark) (B), at temperatures ranging from 18 to 24°C. LD data in (B) are the same as in (A) and included for the purpose of direct comparison. Days to flowering were recorded at the time of appearance of the first open flower. Data points represent the mean ±SE of at least 10 plants for each genotype and condition. (PDF) [file pgen.1006413.s004.pdf]

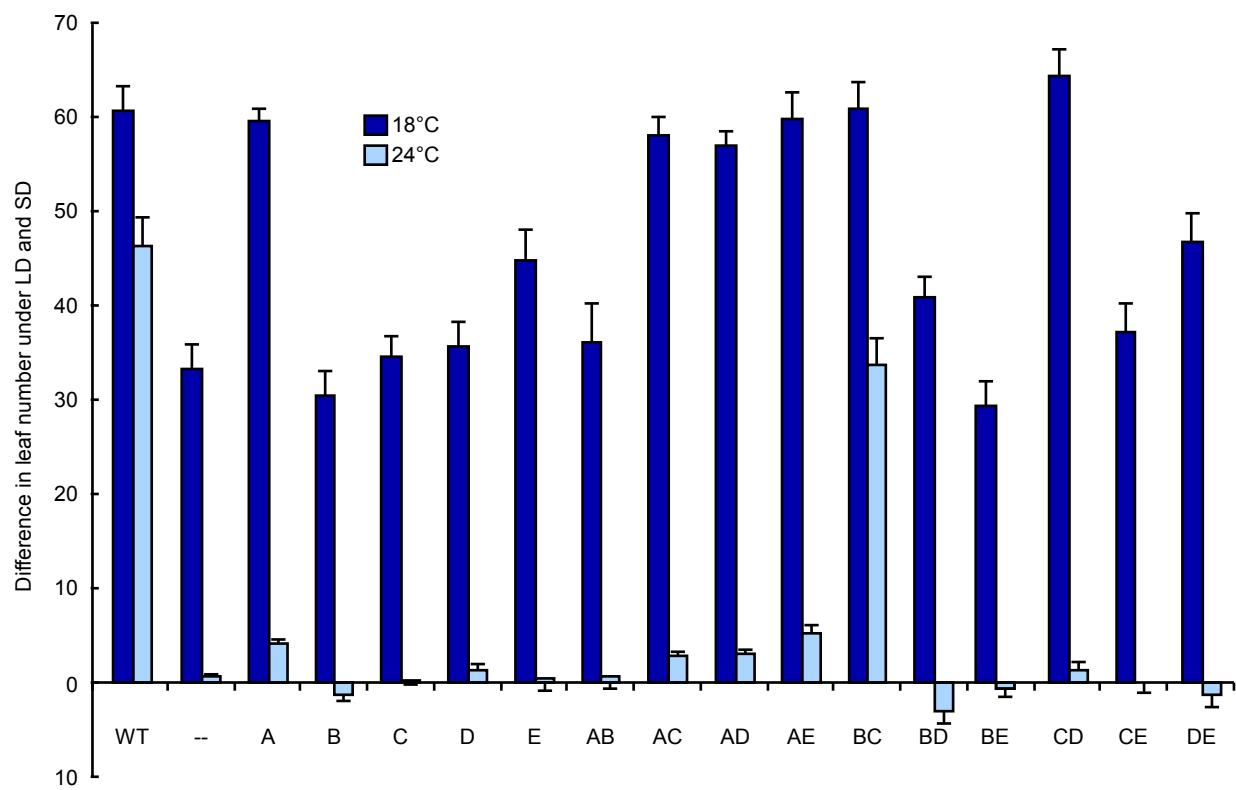

Supplement: S5 Fig — The photoperiodic effect was obtained from data on Fig 2 as the difference between flowering in SD minus flowering in LD for each temperature ±SE. (PDF) [file pgen.1006413.s005.pdf]

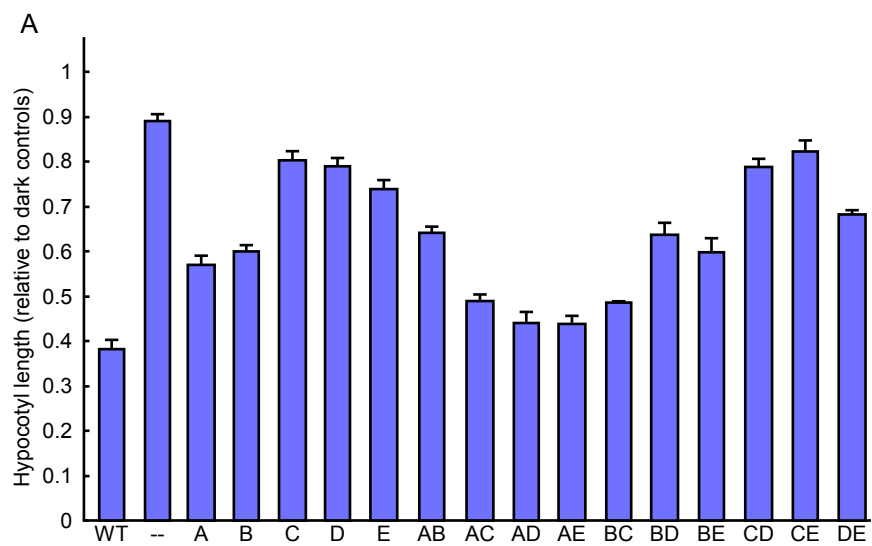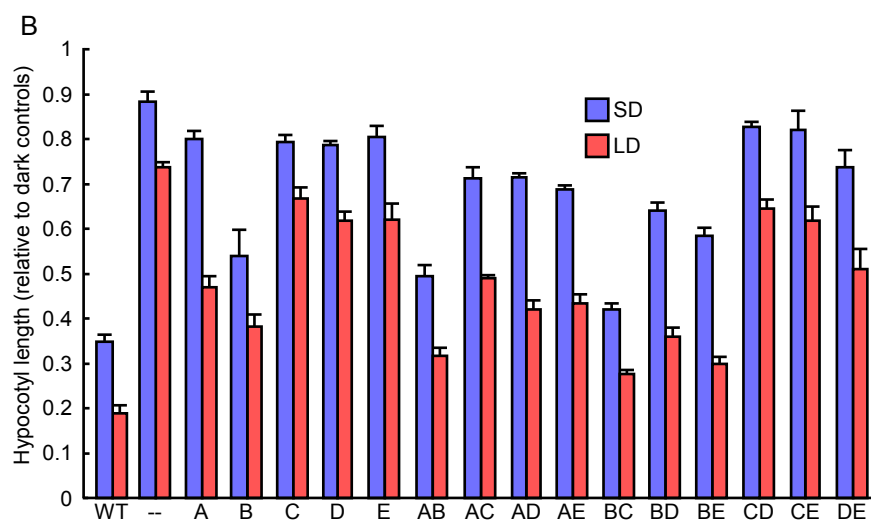

Supplement: S6 Fig — Plants bearing the indicated phytochromes were stratified for 3 days at 4°C in the dark in a solution of 100 μM GA4+7 and then plated on MS salts agar plates and incubated at 23°C either under continuous Blue-light (20 μmol m-2 s-1) (A) under White-light photoperiods (50 μmol m-2 s-1) (B) or kept in darkness (control) for 5 days. Hypocotyls were measured and the values are given relative to the corresponding dark control in each independent experiment. Data are averages ± SE of four independent plates. (PDF) [file pgen.1006413.s006.pdf]

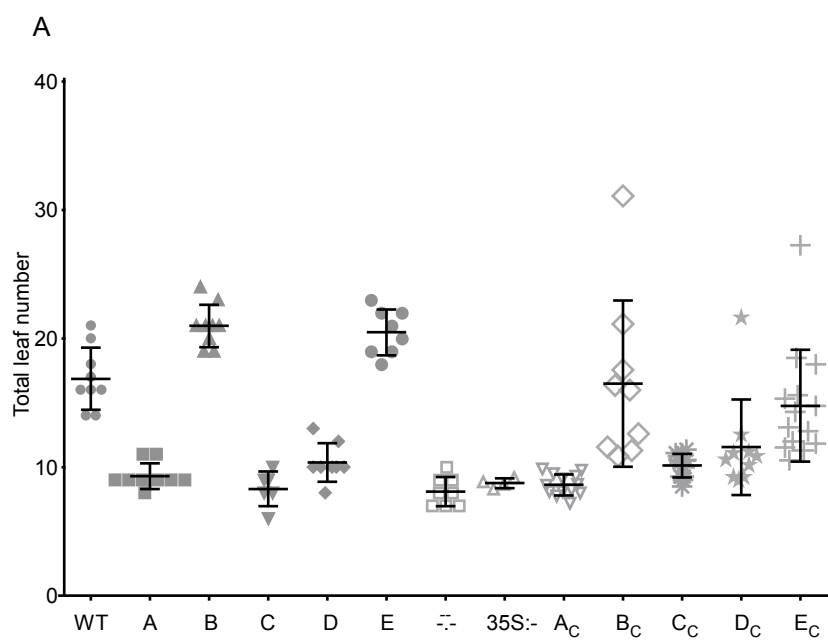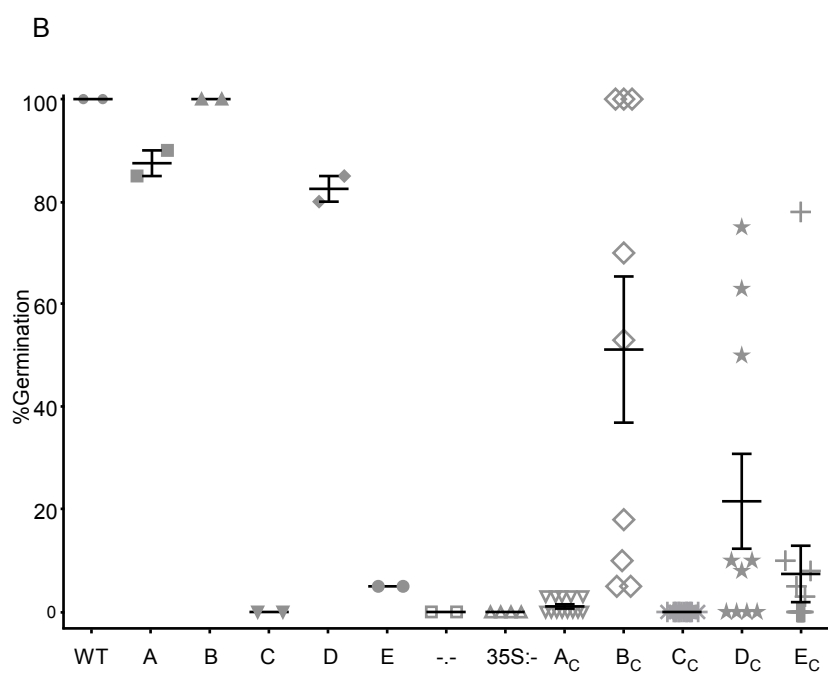

Supplement: S7 Fig — Flowering time (A) and germination rates (B) of independent transgenic lines harboring each phytochrome under the 35S promoter in a background devoid of other phytochromes. Plants harboring the indicated phytochromes were grown under LD conditions at 18°C (A) or under white light at 23°C (B) and total leaf number and germination rates were determined as in Figs 1 and 2. Box plots represent data from 4 transgenic independent lines for the vector control and 13, 8, 14, 10, and 14 independent lines for the constructs bearing 35S:PHYA, 35S:PHYB, 35S:PHYC, 35S:PHYD, and 35S:PHYE, respectively. (PDF) [file pgen.1006413.s007.pdf]

A

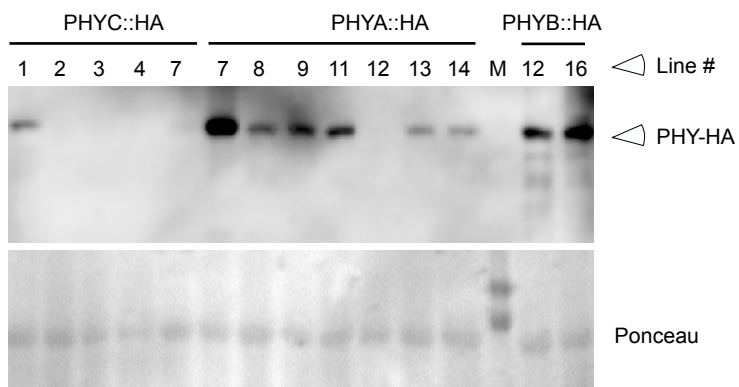

B

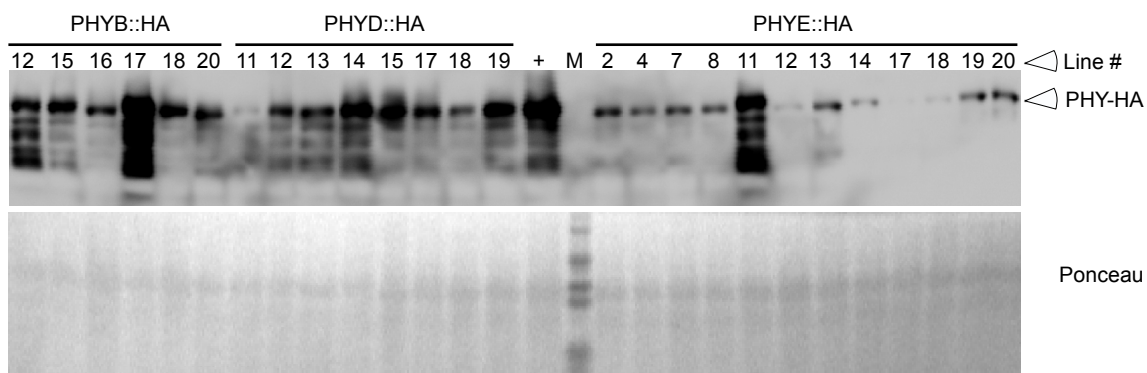

C

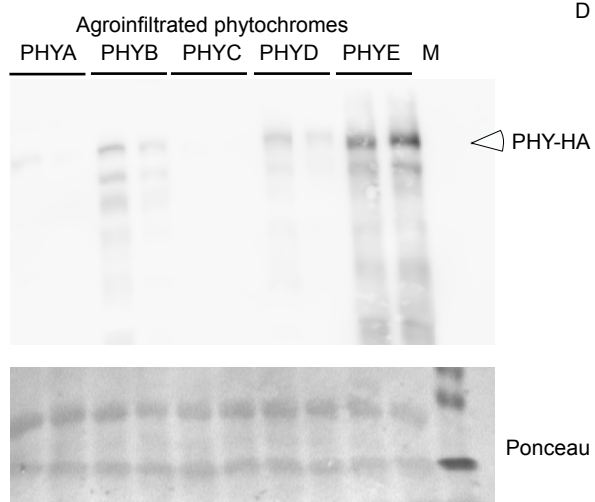

D

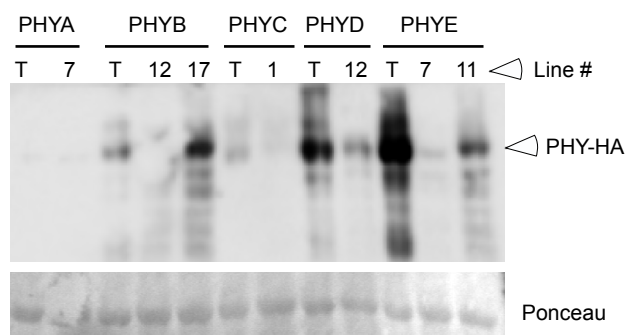

Supplement: S8 Fig — (A, B) Independent transgenic lines for the constructs 35S:PHYA::HA, 35S:PHYB::HA, 35S:PHYC::HA, 35S:PHYD::HA, and 35S:PHYE::HA were grown in the dark for seven days. After grinding, total protein was determined in supernatants and 50 μg of each sample were subjected to SDS-PAGE, and immunoblots detected with anti-HA monoclonal antibodies (Roche 3F10, 2013819). Quantification of bands in (B) are shown in S2 Table. (C, D) Transient expression of constructs 35S:PHYA::HA, 35S:PHYB::HA, 35S:PHYC::HA, 35S:PHYD::HA, and 35S:PHYE::HA in tobacco leaves by agroinfiltration and its comparison to selected Arabidopsis transgenic lines from (A) and (B) is shown in (D). Tobacco plants were kept in the dark for two day before harvest and tobacco extracts are indicated by “T” over each lane. (PDF) [file pgen.1006413.s008.pdf]

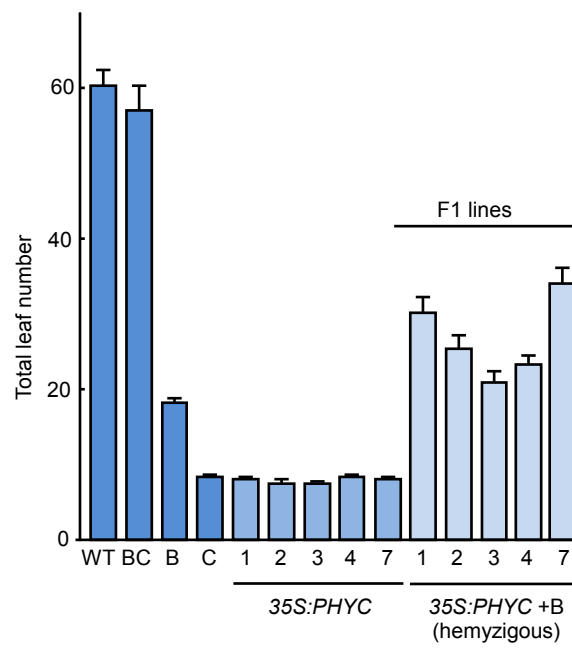

Supplement: S9 Fig — Independent transgenic lines bearing phyC under the 35S promoter were crossed with quadruple phytochrome mutants bearing only phyB. F1 lines were grown under SD at 23°C and the total leaf number was determined as in Fig 2. Data points represent the mean ±SE of at least 12 plants for each genotype. (PDF) [file pgen.1006413.s009.pdf]

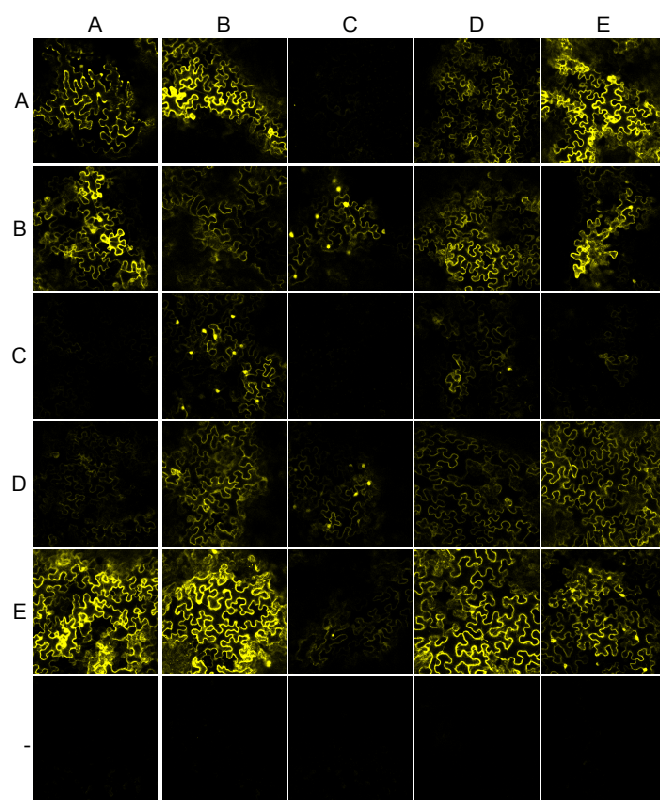

Supplement: S10 Fig — Each possible pair of phytochromes was transiently co-expressed in Nicotiana benthamiana leaves as a fusion to either the N-terminal portion of Enhanced Yellow Fluorescence Protein (nEYFP, indicated on the left) or the C-terminal portion of EYFP (cEYFP, indicated above the panels). Plants remained in the dark for two days before confocal microscopy. Negative controls, nEYFP alone paired with phytochrome-cEYFP are shown in the bottom panels. (PDF) [file pgen.1006413.s010.pdf]

A

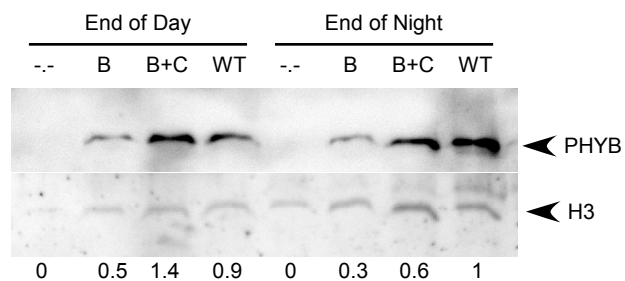

B

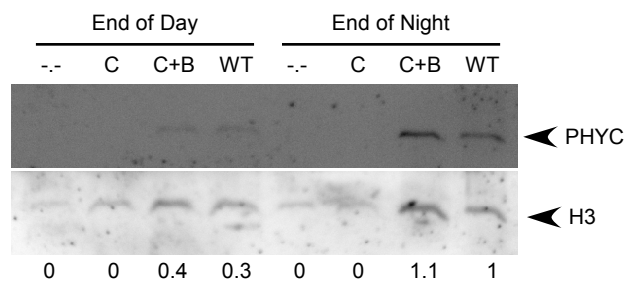

C

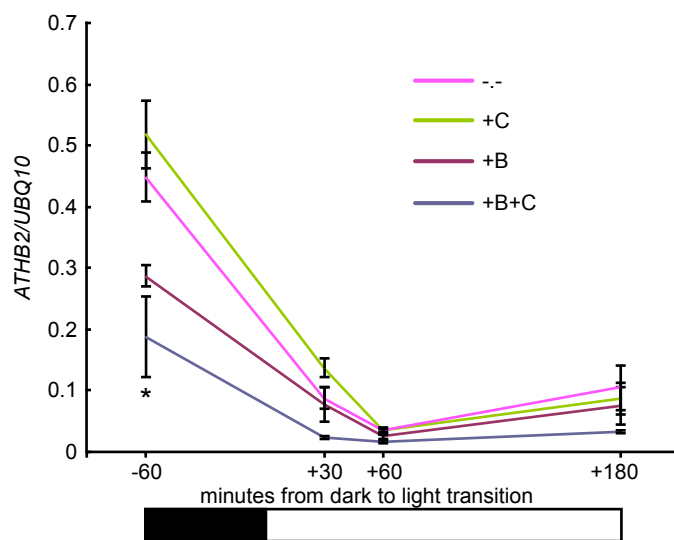

D

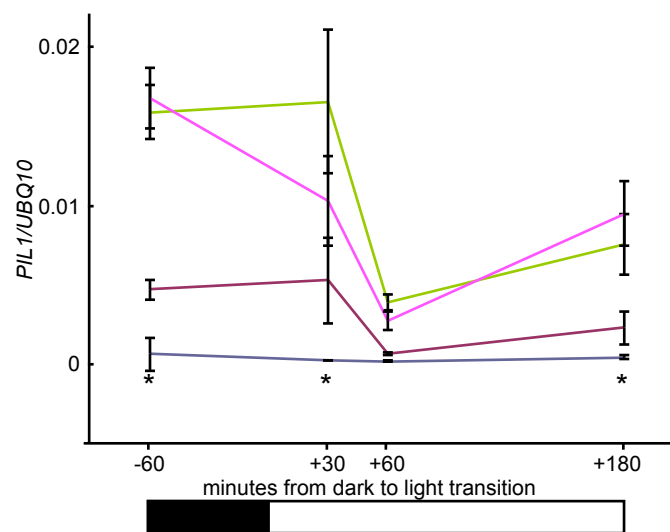

E

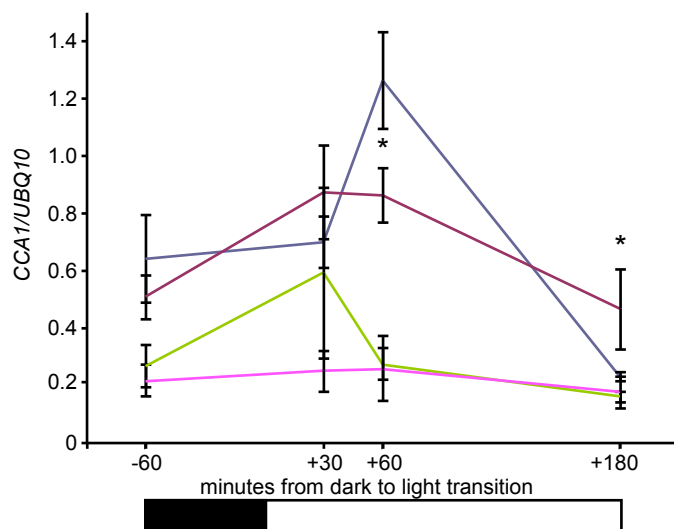

F

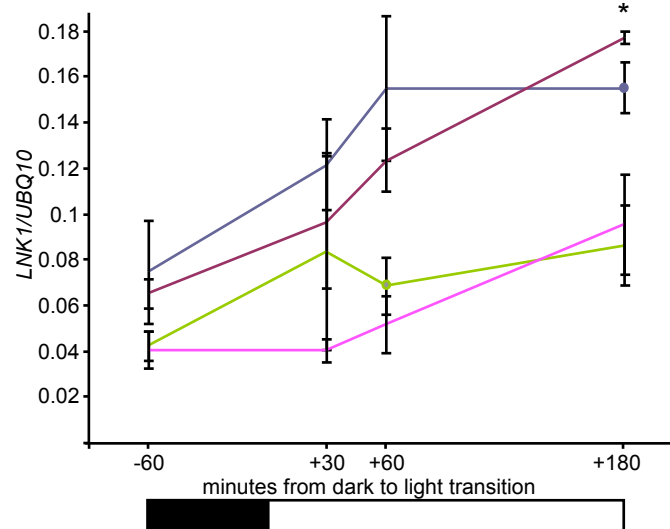

Supplement: S11 Fig — (A-B) Photoreceptor levels in the nucleus during at the end of day and end of night periods. Seedlings of the genotypes bearing only the phytochromes indicated above each lane and the WT control bearing all five phytochromes were grown under SD conditions for 7 days and harvested 1 h before lights-on and 8 h later, 1 h before lights-off. After nuclei enrichment (See Materials and Methods), proteins were detected by immunoblots using either anti phyB (A) or anti phyC (B) monoclonal antibodies. Each band was quantified relative to Histone 3 (bottom panels). Below each lane, the numbers indicate relative quantities of each of PHYB (A) or PHYC (B) apoproteins. (C-F) Coordinated action of phyB and phyC to regulate gene expression during the dark to light transition. Seven day-old seedlings of the indicated genotypes (labels indicate the phytochromes present) were grown in SD (8h light/16 h dark) for seven days and harvested during the dark to light transition as indicated. Transcription levels of shade induced genes ATHB2 (C) and PIL1 (D), and morning-expressed clock genes CCA1 (F) and LNK1 (G) were determined by quantitative real-time PCR, relative to UBQ10 controls. The Error bars represent SE of three biological replicates, and * indicate P < 0.05, by one-way-ANOVA and Tukey contrasts between B and BC. (PDF) [file pgen.1006413.s011.pdf]

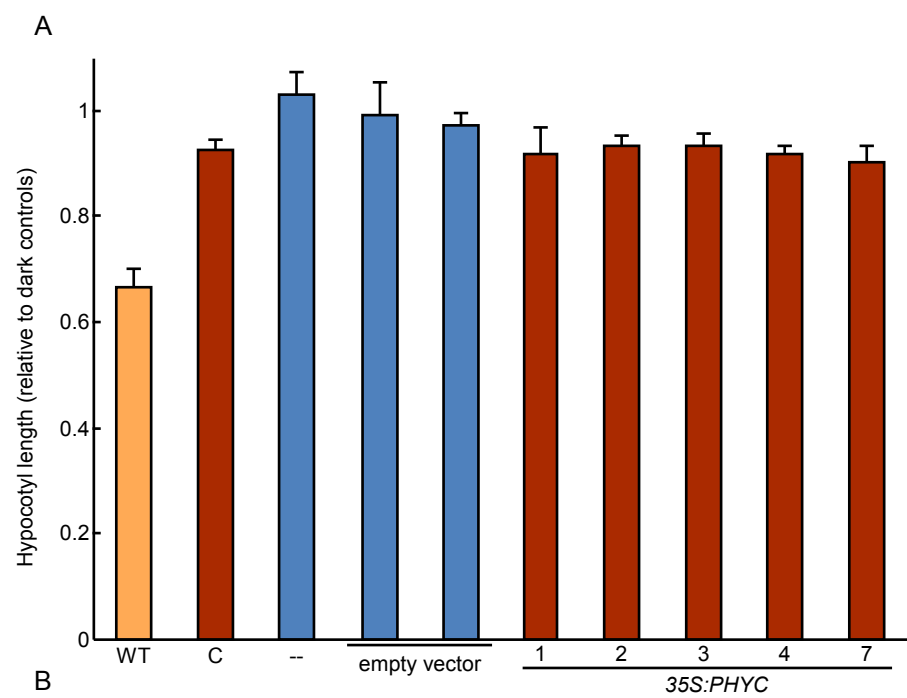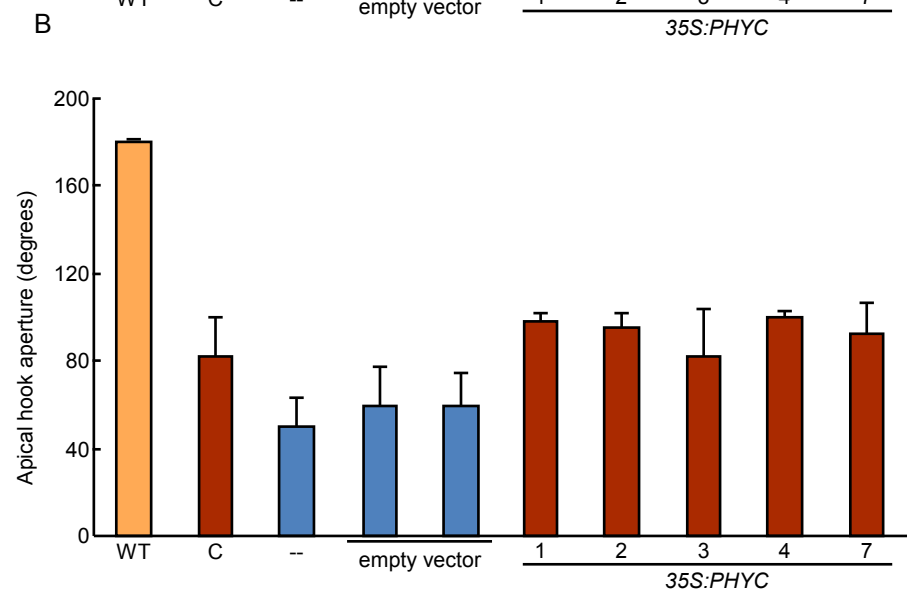

Supplement: S12 Fig — Subtle effects of phyC in the absence of other phytochromes. Phytochromes present are shown. All transgenic lines were generated in the phyA phyB phyC phyD phyE background and the construction utilized is indicated. Seeds were stratified for 3 days at 4°C in the dark in a solution of 100 μM GA4+7, plated on MS agar plates, and incubated at 23°C either under continuous red (R) light (20 μmol m-2 s-1) or kept in darkness (control) for 5 days. (A) Hypocotyls were measured as indicated in Materials and Methods and the values obtained under R are presented relative to the corresponding dark control in each independent experiment. Data are averages ± SE of four independent plates. (B) Apical hook opening was determined as the angle between the apical hook and the hypocotyl, taking the average data value for each genotype in each plate as the experimental unit. Data are averages ± SE of four independent plates. (PDF) [file pgen.1006413.s012.pdf]
