# Supplementary material for: Bottom-up Assembly of the Phytochrome Network
Source: PLoS Genet. 2016 Nov 7;12(11):e1006413. doi: 10.1371/journal.pgen.1006413 (PMC5098793; doi:10.1371/journal.pgen.1006413)
Supplement: S1 Table — (PDF) [file pgen.1006413.s013.pdf]

S1 Table. The sensitivity of triple and quadruple phytochrome mutants to GA during germination.

	Gibberelin (GA_{4+7}) concentration*				
	0 µM	0.1 µM	1 µM	10 µM	100 µM
Wild type	0.0% ± 0.0%	12.8% ± 4.6%	72.8% ± 6.5%	91.3% ± 3.7%	93.1% ± 3.0%
phyABC	0.0% ± 0.0%	3.8% ± 1.3%	40.0% ± 4.7%	82.5% ± 4.4%	94.7% ± 1.3%
phyABD	0.0% ± 0.0%	1.3% ± 0.6%	10.9% ± 2.5%	55.3% ± 6.4%	91.7% ± 1.1%
phyABE	0.0% ± 0.0%	4.1% ± 2.0%	22.5% ± 2.8%	83.4% ± 4.8%	93.1% ± 3.1%
phyACD	5.0% ± 2.8%	23.4% ± 5.2%	79.4% ± 4.2%	92.2% ± 3.8%	99.1% ± 0.5%
phyACE	3.1% ± 1.4%	21.9% ± 5.3%	69.1% ± 3.5%	89.4% ± 2.8%	95.6% ± 1.4%
phyADE	2.5% ± 1.6%	23.1% ± 4.8%	73.4% ± 7.5%	85.6% ± 4.7%	90.3% ± 3.8%
phyBCD	0.0% ± 0.0%	8.1% ± 2.9%	32.5% ± 5.2%	81.3% ± 5.4%	92.5% ± 2.3%
phyBCE	0.0% ± 0.0%	3.4% ± 1.3%	43.8% ± 5.5%	89.4% ± 3.1%	92.2% ± 2.5%
phyBDE	0.3% ± 0.3%	8.4% ± 3.1%	35.9% ± 6.0%	84.7% ± 5.0%	93.7% ± 2.3%
phyCDE	4.7% ± 2.0%	33.4% ± 6.4%	85.6% ± 2.6%	95.3% ± 1.2%	98.4% ± 0.6%
phyBCDE	0.6% ± 0.4%	20.9% ± 3.5%	66.6% ± 4.6%	89.1% ± 3.8%	91.9% ± 3.3%
phyACDE	2.8% ± 1.3%	36.6% ± 5.6%	83.1% ± 3.4%	94.7% ± 2.0%	97.8% ± 1.1%
phyABDE	0.0% ± 0.0%	0.0% ± 0.0%	2.2% ± 1.1%	15.9% ± 3.5%	73.1% ± 4.7%
phyABCE	0.0% ± 0.0%	2.7% ± 1.0%	13.8% ± 2.5%	58.1% ± 7.4%	85.0% ± 3.0%
phyABCD	0.0% ± 0.0%	0.9% ± 0.7%	12.8% ± 3.7%	42.2% ± 6.3%	79.4% ± 4.2%
phyABCDE	0.0% ± 0.0%	0.0% ± 0.0%	1.9% ± 0.8%	26.9% ± 5.4%	70.6% ± 4.9%

**All treatments were done in the presence of a concentration of 100 uM of Paclobutrazol.*
