# Supplementary material for: Bottom-up Assembly of the Phytochrome Network
Source: PLoS Genet. 2016 Nov 7;12(11):e1006413. doi: 10.1371/journal.pgen.1006413 (PMC5098793; doi:10.1371/journal.pgen.1006413)
Supplement: S2 Table — The relative quantification of each band corresponding to tagged versions of phytochrome is included (data obtained from S8B Fig). (PDF) [file pgen.1006413.s014.pdf]

S2 Table. Germination and flowering time of transgenic quintuple phytochrome mutant plants constitutively expressing phyB, phyD or phyE.

| | Germination % | | | Flowering (Total leaves) | | | Relative |
|---|---|---|---|---|---|---|---|
| Line | Mean | ± | SE | Mean | ± | SE | protein levels |
| WT | 100.0% | ± | 0.0% | 16.9 | ± | 0.8 | -.- |
| *phyACDE* | 100.0% | ± | 0.0% | 21.0 | ± | 0.6 | -.- |
| *phyABCE* | 82.5% | ± | 2.5% | 10.4 | ± | 0.5 | -.- |
| *phyABCD* | 5.0% | ± | 0.0% | 20.5 | ± | 0.6 | -.- |
| *phyABCDE* | 0.0% | ± | 0.0% | 8.1 | ± | 0.4 | -.- |
| 35S::(-)#1 | 0.0% | ± | 0.0% | 9.2 | ± | 0.4 | -.- |
| 35S::(-)#3 | 0.0% | ± | 0.0% | 8.7 | ± | 0.5 | -.- |
| 35S::(-)#5 | 0.0% | ± | 0.0% | 8.3 | ± | 0.3 | -.- |
| 35S::(-)#6 | 0.0% | ± | 0.0% | 8.9 | ± | 0.6 | -.- |
| 35S::PHYB#12 | 100.0% | ± | 0.0% | 21.1 | ± | 1.8 | 1.00 |
| 35S::PHYB#15 | 100.0% | ± | 0.0% | 16.4 | ± | 1.0 | 1.40 |
| 35S::PHYB#16 | 5.0% | ± | 0.0% | 11.6 | ± | 0.5 | 0.84 |
| 35S::PHYB#17 | 100.0% | ± | 0.0% | 31.1 | ± | 1.0 | 5.98 |
| 35S::PHYB#18 | 17.5% | ± | 7.5% | 11.3 | ± | 0.6 | 1.47 |
| 35S::PHYB#20 | 52.5% | ± | 2.5% | 17.6 | ± | 1.4 | 1.16 |
| 35S::PHYD#11 | 62.5% | ± | 2.5% | 9.3 | ± | 0.7 | 0.09 |
| 35S::PHYD#12 | 10.0% | ± | 0.0% | 12.5 | ± | 1.1 | 0.70 |
| 35S::PHYD#13 | 50.0% | ± | 40.0% | 9.3 | ± | 0.7 | 1.27 |
| 35S::PHYD#14 | 75.0% | ± | 5.0% | 21.7 | ± | 1.8 | 1.39 |
| 35S::PHYD#15 | 0.0% | ± | 0.0% | 9.0 | ± | 0.5 | 1.09 |
| 35S::PHYD#17 | 0.0% | ± | 0.0% | 11.1 | ± | 0.3 | 0.72 |
| 35S::PHYD#18 | 7.5% | ± | 2.5% | 10.2 | ± | 0.7 | 0.36 |
| 35S::PHYD#19 | 10.0% | ± | 5.0% | 11.2 | ± | 0.7 | 1.71 |
| 35S::PHYE#2 | 10.0% | ± | 5.0% | 11.3 | ± | 0.6 | 0.53 |
| 35S::PHYE#4 | 0.0% | ± | 0.0% | 11.6 | ± | 0.5 | 0.24 |
| 35S::PHYE#7 | 0.0% | ± | 0.0% | 14.8 | ± | 0.5 | 0.37 |
| 35S::PHYE#8 | 0.0% | ± | 0.0% | 11.9 | ± | 0.5 | 0.40 |
| 35S::PHYE#11 | 77.5% | ± | 2.5% | 27.3 | ± | 1.3 | 1.85 |
| 35S::PHYE#12 | 0.0% | ± | 0.0% | 12.8 | ± | 1.0 | 0.14 |
| 35S::PHYE#13 | 0.0% | ± | 0.0% | 18.5 | ± | 2.1 | 0.43 |
| 35S::PHYE#14 | 0.0% | ± | 0.0% | 15.4 | ± | 1.1 | 0.13 |
| 35S::PHYE#17 | 0.0% | ± | 0.0% | 13.1 | ± | 0.7 | 0.02 |
| 35S::PHYE#18 | 0.0% | ± | 0.0% | 14.3 | ± | 1.2 | 0.02 |
| 35S::PHYE#19 | 7.5% | ± | 2.5% | 18.0 | ± | 0.6 | 0.31 |
| 35S::PHYE#20 | 5.0% | ± | 5.0% | 15.6 | ± | 0.7 | 0.45 |

WT: Wild type; 35S: 35S CaMV promoter
