# Supplementary material for: Bottom-up Assembly of the Phytochrome Network
Source: PLoS Genet. 2016 Nov 7;12(11):e1006413. doi: 10.1371/journal.pgen.1006413 (PMC5098793; doi:10.1371/journal.pgen.1006413)
Supplement: S3 Table — (PDF) [file pgen.1006413.s015.pdf]

S3 Table. Primers used in this work

S3A Table. Primers used to clone phytochromes cDNAs.

Locus	Primer	Secuence	
phyA	pCHF5-PHYA-fd1(BamHI)	cgcggatccATGTCAGGCTCTAGGCCGACTCA	
	pCHF5-PHYA-fd2	GCGTTCTGTGCATCTTGCTAA**C**CTAGA	Silent mutation that eliminates XbaI site
	pCHF5-PHYA-rv1	GGTATGCGTTAACCTGATCTCTAG**G**TTAGCA	
	pCHF5-PHYA-rv2(XbaI-SalI)	tgctctagagtcgacCTTGTTTGCTGCAGCGAGTTCCG	
phyB	CHF5-PHYB-Fd1(BamHI)	gccggatccATGGTTTCCGGAGTCGGGGGTA	
	CHF5-PHYB-Rv1	GTGGAGTCTTGCGTC**A**ACGGTGTAC	Silent mutation that eliminates SalI site
	CHF5-PHYB-Fd2	GTACACCGT**T**GACGCAAGACTCCAC	
	CHF5-PHYB-Rv2	CCAGTATTCTTGGAATG**A**ATCCAAACCGGATT	Silent mutation that eliminates BamHI site
	CHF5-PHYB-Fd3	CCTTGTTAAATCCGGTTTGGAT**T**CATTCCAAGA	
	CHF5-PHYB-Rv4(XbaI-SalI)	tgctctagagtcgacATATGGCATCATCAGCATCATGTCACC	
phyC	pCHF5-PHYC-fd1(BamHI)	cgcggatccATGTCATCGAACACTTCACGAAGCTGTTCTACTAGATC**C**AGACA	Silent mutation that eliminates XbaI site
	pCHF5-PHYC-rv1	GTTTCCGTGTAGCTTTGCGTC**A**ACGAG	Silent mutation that eliminates SalI site
	pCHF5-PHYC-fd2	GAGTTTCTTCACAAGTTCTCGT**T**GACG	
	pCHF5-PHYC-rv2(XbaI-SalI)	tgctctagagtcgacAATCAAGGGAAATTCTGTGAGGATCACAAAGG	
phyD	pCHF5-PHYD-Fd1(BamHI)	cgcggatccATGGTCTCCGGAGGTGGTAGCAA	
	pCHF5-PHYD-Rv1	GCGTGGAGTCTCGCGTC**A**ACAGT	Silent mutation that eliminates SalI site
	pCHF5-PHYD-Fd2	GCTATTCAACAGTACACTGT**T**GACGCGA	
	pCHF5-PHYD-Rv2	GCCACCGCGTTGGAT**A**CGGGAGA	Silent mutation that eliminates BamHI site
	pCHF5-PHYD-Fd3	AGATCACAGCTTATCTCTCCCG**T**ATCCA	
	pCHF5-PHYD-Rv3	TGCCTCCTCTGAACTTC**G**AGAGCT	Silent mutation that eliminates XbaI site
	pCHF5-PHYD-Fd4	GAGCTGCAGCAAGCTCT**C**GAAGT	
	pCHF5-PHYD-Rv4(XbaI-SalI)	tgctctagagtcgacTGAAGAGGGCATCATCATCATTAGAGGAACC	
phyE	pCHF5-PHYE-Fd1(BamHI)	cgcggatccATGGGATTCGAGAGTTCAAGCTCAGC	
	pCHF5-PHYE-Rv1(XbaI-SalI)	tgctctagagtcgacCTTTATGCTTGAACTACCCTCTGTTCCTC	

*Start codon
BamHI restriction site
XbaI restriction site
SalI restriction site
Bold indicates a point mutation.
CAPITAL LETTERS indicate coding sequence

S3B Table. Primers used for qRT-PCR

**Primer**	**Sequence**	**Product**
UBQ10-1	TCAATTCTCTCTACCGTGATCAAGATGCA	318 pb
UBQ10-2	GGTGTCAGAACTCTCCACCTCAAGAGTA	
ATHB2-RT-Fd1	GAGGAATCGACGTGAACAGACCAC	272 pb
ATHB2-RT-Rv1	CAATGCTTGCTTCTGCTTCGGAT	
PIL1-RT-Fd1	GGACCCTTCAACTTCAAGTTCAGA	153 pb
PIL1-RT-Rv1	TAGGCAGGAATTGCGGTATTGA	
CCA1-RT-Fd1	CTGTGTCTGACGAGGGTCGAA	167 pb
CCA1-RT-Rv1	AACTGGTGTTAACTGAGCTGTGA	
LNK1-RT-Fd1	ATCTCATCCAATGATTCCTCAGC	152 pb
LNK1-RT-Rv1	GCTTCAGCAATGAAAGAGCCA	
