# Supplementary material for: Bottom-up Assembly of the Phytochrome Network
Source: PLoS Genet. 2016 Nov 7;12(11):e1006413. doi: 10.1371/journal.pgen.1006413 (PMC5098793; doi:10.1371/journal.pgen.1006413)
Supplement: S1 Text — (DOC) [file pgen.1006413.s016.doc]

# Supporting text

**Supporting materials and Methods**

# Plant Material and Growth Conditions. *phyA-211*; *phyB-9*, *phyC-2*, *phyD-201* and *phyE-201* alleles are in the Columbia background. Segregating populations were genotyped as previously described to identify triple and quadruple mutants.

For experiments with seedlings, sterilized seeds were suspended in 100 μM GA_4+7_ (Duchefa Biochemie, Haarlem, The Netherlands), stratified for 3 days at 4ºC and then pipetted on plates with Murashige Skoog Salts media and 0.8 % Plant Agar (Duchefa Biochemie). Light treatments were performed on dedicated growth chambers (Model I30BLL, Percival Scientific, Perry, IA, U.S.A.). For red, far-red and blue, we used light-emitting diodes. For the hypocotyl measurements assays, 15 seeds were plated for each replicate, being the value of each replicate the average of the 10 tallest seedlings.

For germination assays, sterilized seeds were directly plated on MS salts plates (0.8 % agar) and given a post-imbibition saturating 5 min FR pulse to revert seed phytochrome to the Pr form. Then, stratified in the dark for 3 days at 4ºC. After stratification, seeds were incubated at 23ºC for 6 days under the indicated light regimes, before counting germinated seeds (radicle emergence).

**Generation of transgenic plants.** The phytochrome cDNAs were all obtained by retrotranscription from the Col-0 accession RNA, and finally cloned in the pCHF5 plasmid fused to the C-terminus HA tag. Homozygous T3 lines derived from T2 lines showing 3:1 segregation for the resistance to DL-Phosphinothricin (BASTA, Duchefa Biochemie), indicative of single locus insertions, were used in all experiments, except when otherwise stated.

# Cloning. The complete coding sequence of each Arabidopsis phytochrome was PCR-amplified from cDNA obtained by retrotranscription of total RNA isolated from wild type Col-0 seedlings. In the case of phyA, phyB, phyC and phyD, some restriction sites were eliminated by introducing silent mutations by using PCR with specific primers (S3A Table). Fragments were then assembled together by fusion-PCR, and restriction sites added to both 5´and 3´ends. Each cDNA was cloned in the pBlueScript plasmid as a BamHI-XbaI fragment and the sequence confirmed by Sanger sequencing. Finally, each fragment was subcloned in the corresponding binary vector, as a BamHI-SalI fragment. The pCHF5 vector was used for constitutive expression under the CAMV 35S promoter of phytochrome fusions to the HA tag (phytochrome C-terminal fusions). For BiFC experiments, phytochromes were subcloned in the binary vectors pCardo1-C-nEYFP and pCardo1-C-cEYFP (this work). EYFP sequences were derived from pSAT6-nEYFP-N1 and pSAT6-cEYFP-N1plasmids ([**https://www.bio.purdue.edu/people/faculty/gelvin/nsf/protocols_vectors.htm**](https://www.bio.purdue.edu/people/faculty/gelvin/nsf/protocols_vectors.htm)). For co-localization assays, the corresponding phytochromes were subcloned in the pCardo1-C-GFP5 and pCardo2.1-C-Cerulean, for GFP or cerulean C-terminal fusions.

**Bimolecular Fluorescence Complementation assay**.

*Agrobacterium tummefaciens* (GV3301) containing each phytochrome tagged to the nEYFP or cEYFP, the pBIN19-35S-P19 and the pCardo2.1-ECFP-NSL were cultivated O.N. in 20 ml of LB media containing the corresponding antibiotics, 20 mM MES pH5.6 and 0.1 mM Acetosyringone. When cultures reached an O.D. of 1, they were centrifuged and the pellet was resuspended in 20 mM MES pH5.6, 10 mM MgCl_2_ and 0.12 mM Acetosyringone. After 3-5 hours at room temperature (about 23-28ºC) to strongly induce virulence, different combinations of the cultures were co-infiltrated in a 1:1 ratio in the leaves of *Nicotiana bentamiana* plants grown under LD at 23 °C. The next day, the plants were either moved to darkness for another two days or treated as indicated in each experiment before imaging. The confocal images were taken using a Zeiss LSM 710 microscope. EYFP was excited at 514 nm and observed at 520-539 nm, whereas ECFP was excited at 458 nm and observed at 466-480 nm.

When it was necessary to fix the leaves of *Nicotiana benthamiana*, the leaves were treated for 15 minutes with a 1.5% formaldehyde solution in water, and then thoroughly washed with water.

**Co-localization assays**

Co-localization assays were performed in the same way than the BiFC experiments.

The confocal images were taken using a Zeiss LSM 710 microscope. GFP5 was excited at 485 nm and observed at 520-539 nm, while Cerulean was excited at 458 nm and observed at 466-480 nm.

**Protein Extraction and Immunoblots**

Protein extracts were prepared by resuspending ground tissue in cold extraction buffer (50 mM Tris-HCl, pH 7.4, 150 mM NaCl, 0.1% [p/v] Nonidet P-40, and 10% glycerol) at a ratio of 1 g tissue mL−1 extraction buffer and then centrifuged at 13,000*g* for 30 min. Aliquots were stored at −80°C to quantify proteins by the method of [Lowry et al. (1951)](http://www.plantphysiol.org/content/160/3/1662.long#ref-30). When used for gel loading, 1 volume of 3× SDS-Urea sample buffer was added to each extract and boiled for 5 min. 50 μg of protein per sample was subjected to 9% SDS-PAGE and transferred to a nitrocellulose membrane (Hybond-ECL; Amersham Biosciences; RPN303D). For phyC, phyD and phyE, the total protein loaded per lane was increased to 100 μg in thicker gels, to facilitate detection by specific monoclonal antibodies.

The HA tag was detected with anti-HA peroxidase (Roche 3F10, 2013819) at a dilution of 1:1000 or with polyclonal anti-HA (Sigma, H6908) at a dilution of 1:500 followed by anti-rabbit IgG peroxidase (Sigma, A0545), at a dilution of 1:6000, as a secondary antibody.

Individual phytochromes were detected using monoclonal antibodies as described in .

**qRT-PCR assays**

100 mg of 7 days seedlings were frozen in liquid nitrogen, and total RNA was extracted using the Spectrum^TM^ Plant total RNA kit (Sigma). 1 μg of total RNA was used to synthesize cDNA with M-MLV reverse transcriptase (Invitrogen), and used to quantitate UBQ10, ATHB2, PIL1, CCA1 and LNK1 expression with the LC480II real-time PCR system. The sequences of the primers used are listed on S3B Table.

**References**

1. Strasser, B., et al. (2010). Arabidopsis thaliana life without phytochromes*.* Proc. Natl. Acad. Sci. U. S. A. *107*, 4776-4781.

2. Hirschfeld, M., et al. (1998). Coordination of phytochrome levels in phyB mutants of Arabidopsis as revealed by apoprotein-specific monoclonal antibodies*.* Genetics *149*, 523-535.
